# *In vivo* Imaging Technologies to Monitor the Immune System

**DOI:** 10.3389/fimmu.2020.01067

**Published:** 2020-06-02

**Authors:** Claire E. McCarthy, Jordan M. White, Nerissa T. Viola, Heather M. Gibson

**Affiliations:** Department of Oncology, Karmanos Cancer Institute, Wayne State University, Detroit, MI, United States

**Keywords:** imaging, magnetic resonance imaging (MRI), computed tomography (CT), scintigraphy, optical imaging (OI), ultrasound, positron emission tomography (PET), single-photon emission computed tomography (SPECT)

## Abstract

The past two decades have brought impressive advancements in immune modulation, particularly with the advent of both cancer immunotherapy and biologic therapeutics for inflammatory conditions. However, the dynamic nature of the immune response often complicates the assessment of therapeutic outcomes. Innovative imaging technologies are designed to bridge this gap and allow non-invasive visualization of immune cell presence and/or function in real time. A variety of anatomical and molecular imaging modalities have been applied for this purpose, with each option providing specific advantages and drawbacks. Anatomical methods including magnetic resonance imaging (MRI), computed tomography (CT), and ultrasound provide sharp tissue resolution, which can be further enhanced with contrast agents, including super paramagnetic ions (for MRI) or nanobubbles (for ultrasound). Conjugation of the contrast material to an antibody allows for specific targeting of a cell population or protein of interest. Protein platforms including antibodies, cytokines, and receptor ligands are also popular choices as molecular imaging agents for positron emission tomography (PET), single-photon emission computerized tomography (SPECT), scintigraphy, and optical imaging. These tracers are tagged with either a radioisotope or fluorescent molecule for detection of the target. During the design process for immune-monitoring imaging tracers, it is important to consider any potential downstream physiologic impact. Antibodies may deplete the target cell population, trigger or inhibit receptor signaling, or neutralize the normal function(s) of soluble proteins. Alternatively, the use of cytokines or other ligands as tracers may stimulate their respective signaling pathways, even in low concentrations. As *in vivo* immune imaging is still in its infancy, this review aims to describe the modalities and immunologic targets that have thus far been explored, with the goal of promoting and guiding the future development and application of novel imaging technologies.

## Introduction

The immune system and its functions are complex and dynamic, and imaging provides a unique opportunity to non-invasively monitor these processes *in vivo*. There are a variety of human conditions and diseases that could benefit from immunoimaging. Current cancer immunotherapies are designed to either directly target specific tumor-associated antigens, modulate components of immune activation, or suppress signaling pathways to enhance immune activity. Although responders often show durable disease control, most patients fail to exhibit any benefit. Treatment efficacy is assessed through tumor volume measurements, post-treatment tissue biopsies, or peripheral blood assays, each of which have their limitations. Changes in tumor size may prove misleading, as an influx of helpful immune cells to the microenvironment often contribute to increased volume, a phenomenon termed pseudoprogression (1). Post-therapy biopsy is dependent on accessibility of the tumor, often fails to account for tumor heterogeneity, and is invasive in nature, potentially affecting the neighboring tumor microenvironment and hindering patient consent (2). Peripheral blood assays are currently non-standardized, often require knowledge of specific tumor antigen(s), and may not reflect immune activity within the tumor (3). Thus, there is an imminent clinical need for diagnostic and predictive methods to detect anti-tumor immunity. Aside from cancer, additional immune-associated conditions may clinically benefit from imaging, including autoimmunity, inflammatory disease, and infection or sepsis. These conditions can also utilize imaging to monitor treatment response and/or disease progression by targeting a specific cell type, biomarker, or general inflammatory molecule. This review provides a compilation of current imaging targets and modalities for monitoring immune trafficking and activity in cancer and inflammatory conditions.

Imaging modalities are subcategorized as either anatomical or molecular. Anatomical imaging modalities can be performed with contrast to enhance disease detection at the tissue site, and include magnetic resonance imaging (MRI), computed tomography (CT), and ultrasound. Alternatively, molecular imaging modalities provide functional insight, and include several two- and three-dimensional options. Scintigraphy, or planar gamma imaging, generates a two- dimensional image. Positron emission tomography (PET), single-photon emission computed tomography (SPECT), optical imaging, and photoacoustic, are three-dimensional imaging strategies. PET, SPECT, scintigraphy, ultrasound, MRI, and CT are currently utilized for immune monitoring. However, each modality brings its own unique features that may be more favorable depending on the context. Additionally, the utilization of different imaging mechanisms has led to hybrid imaging such as SPECT/CT, PET/CT, and PET/MRI. Hybrid imaging provides the benefit of molecular sensitivity and spatial resolution fused with anatomical specificity.

Radioisotopes are utilized for PET, SPECT, and scintigraphy. PET isotopes emit positrons, which annihilate electrons and produce two coincident 511 keV photons traveling opposite of each other (180°) that are detected by the PET scanner (4). Both the timing and location of detection for the two photons are critical in determining positional information and image reconstruction. SPECT imaging, a three-dimensional form of scintigraphy, directly detects gamma rays at energies inherent in each isotope, as opposed to the 511 keV gamma rays detected by PET. An in-depth comparison of PET and SPECT is provided in “PET vs. SPECT: strengths, limitations, and challenges” by Rahmim and Zaidi (5), in addition to the works of Vaquero and Kinahan (6), and Reddy and Robinson (7). Alternatively, scintigraphy generates two-dimensional images using a gamma camera, similar to SPECT. However, in contrast to SPECT, planar scintigraphy does provide spatial localization of large, complex tissue morphologies. An overview of candidate PET, SPECT, and scintigraphy radioisotopes and their properties are shown in Table 1 (8–24).

**Table 1:** An overview of PET, SPECT, and scintigraphy radioisotopes and their metal properties, half-life, and methods for labeling are provided for consideration of tracer development.

**Modality**	**Radioisotopes**		**Half-life**	**Method of labeling**	**References**
PET	Non-metal	^11^C	20.4 min	[^11^C]methyl Iodide, [^11^C]methyl Triflate, Choline	(19)
		^18^F	109.7 min	*N*-hydroxy-succinimidyl ^18^F-fluorobenzoate, click chemistry, ^18^F-aluminum NOTA	(8)
		^76^Br	16.2 h	Direct bromination, bromine-labeled activated esters	(9)
		^124^I	4.18 d	Direct labeling	(8, 9)
	Metal	^68^Ga	67.7 min	DOTA, NOTA	(8, 17)
		^44^Sc	3.97 h	DOTA	(20)
		^64^Cu	12.7 h	NOTA, DOTA, TETA	(8, 9, 17, 18)
		^86^Y	14.7 h	DOTA, DTPA	(8, 9, 18)
		^55^Co	17.5 h	DOTA, HBED, TETA, NOTA	(9, 18)
		^72^As	25.9 h	Direct labeling, Trithiol containing chelators and dithiol lipoic acid	(9, 18)
		^89^Zr	3.27 d	DFO	(8, 9, 17, 18)
SPECT/ scintigraphy	Non-metal	^123^I	13.22 h	Direct labeling	(21–24)
		^131^I	8.02 d	Direct labeling	(8, 10)
	Metal	^99m^Tc	6.02 h	Diamide dithiols, triamide thiols, mapt, MAP, HyNic	(11–14)
		^111^In	2.8 d	H_4_Octapa	(15)
		^67^Ga	3.26 d	DFO, NOTA, DOTA	(16)
		^177^Lu	6.65 d	DOTA	(8)

*Of note, metal radionuclides can residualize, or remain, in the cell if their biological molecule is internalized. Non-metal radionuclides do not residualize. Residualizing radionuclides are beneficial for RIT and increased tumor-to-background ratios. DOTA, 1,4,7,10-tetraazacyclododecane-1,4,7,10-tetraacetic acid; NOTA, 1,4,7-triazacyclononane-1,4,7-triacetic acid; TETA, triethylenetetramine; DTPA, diethylenetriamine pentaacetic acid; HBED, N,N′-bis (2-hydroxybenzyl) ethylenediamine-N,N′-diacetic acid; DFO, desferrioxamine; MAP, 2,3-bis(mercaptoacetamido)propanoate; mapt, 4,5-bis(thioacetamido)pentanoate; HyNic, 6-hydrazinonicotinamide*.

For PET, SPECT, and scintigraphy tracer development, the half-life (t_1/2_) of the radioisotope should be comparable with the biological t_1/2_ of the tracer. The fate of the radionuclide after tracer delivery to the disease site is important. Typically, radiometals are retained, or residualized, in the lysosomal compartment of cells even after the peptide and antibody conjugates are proteolytically degraded (9). Prolonged intracellular accumulation of the radioactivity can improve signal after clearance of the radiotracer from non-specific tissues (7). Attachment of the radionuclide via linker, chelate, or prosthetic group should provide stability to prevent detachment, without affecting the binding ability of the tracer to its target. The commonly used chelators and precursors for prosthetic groups are commercially available and have been extensively discussed in various reviews (17, 25, 26). Additionally, the dose exposure to the patient is a concern; a balance must be sought between the lower-dose shorter-lived radionuclides vs. the benefit of increased signal to noise ratios provided by longer-lived radionuclides (27).

Ultrasound is a readily available and inexpensive imaging modality that provides real-time images for evaluation of blood vessels, tissue, and organs (28). Ultrasound technology emits high-frequency sound waves into tissue. An anatomic image is then produced from detection of sound waves either transmitted through the tissue or echoed back. MRI takes advantage of the high composition of hydrogen protons in tissue, detecting magnetic field-induced signals to achieve tissue resolution, which can be further enhanced with the use of a contrast agent (29). MRI contrast agents are typically paramagnetic metal complexes [containing either gadolinium (III), dysprosium (III), or manganese (II)] or superparamagnetic agents such as iron oxide nanoparticles (SPION) and ultrasmall superparamagnetic iron oxide (USPIO). Gadolinium represents the most commonly used class of MRI contrast agents, however SPIONs provide better contrast and higher sensitivity specifically for detecting inflammation (30, 31). MRI has high anatomic resolution and excels in soft-tissue contrast images.

CT imaging is a compilation of multiple x-ray transmissions to reconstruct high-resolution images (32). The anatomical contrast of a CT is produced by the attenuation of the x-rays as they pass through, or are deflected by, tissue. However, there are instances in which improved contrast is required, therefore contrast agents are utilized to improve imaging. One example of CT contrast agents are iodinated compounds which improve intravascular CT contrast (33). Both PET and SPECT imaging are often co-registered with CT to provide accurate anatomic localization of the probe (4, 34, 35).

**Figure 1:**
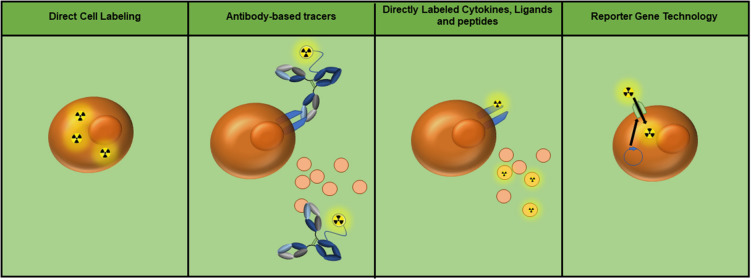
Currently utilized whole-body immune imaging technologies. Depending on the imaging modality, radioactive symbols may be interchanged with the appropriate tag (i.e., fluorescence, superparamagnetic iron oxide nanoparticles, nanobubbles, etc.). **(Left to right)** Cells can be directly labeled to track their location and migration. Antibody-based tracers can target molecules or receptors on the cell surface, as well as soluble protein such as cytokines. Proteins including cytokines, ligands, and peptides can also be directly labeled to detect cell populations expressing their receptors. Finally, reporter gene technology can be utilized to achieve specific labeling of adoptive cell populations (e.g. expression of the sodium/iodide symporter as a means of ^99m^Tc pertechnetate uptake).

Each of the discussed imaging modalities have been tested in some capacity to evaluate immune cell presence and/or function. Promising new adoptive cell therapies (ACT) for cancer, including *ex vivo* expanded tumor-infiltrating lymphocytes (TILs) and chimeric antigen receptor T cells (CAR-T cells), would benefit from imaging technologies that track cell fate *in vivo*. This can be achieved by labeling the T cells with an imaging agent prior to patient infusion or by the inclusion of a reporter gene. A similar approach is used to identify the location of inflammation, in which macrophages are loaded with iron oxide nanoparticles (Fe NP) and their accumulation is detected by MRI. The use of tracers to target cell surface markers, checkpoint, or costimulatory molecules, and/or secreted cellular products can identify localization and/or function of specific immune cell populations (Figure 1). Studies evaluating each of these modalities are discussed.

## Metabolic and Indirect Physiologic Imaging

As a means of imaging metabolically active cells, the radioactive ^18^F labeled glucose analog [^18^F]fluorodeoxyglucose (^18^F-FDG) is commonly utilized with PET imaging, particularly in oncology to detect glucose-consuming tumors (36, 37). Given the high glycolytic activity of stimulated immune cells, the use of ^18^F-FDG-PET has been tested for determination of response to tumor immunotherapy (38, 39). These studies report an increase in tumor-localized ^18^F-FDG uptake after immunotherapy, which associates with response. However, ^18^F-FDG is unable to distinguish metabolic uptake between tumor vs. immune cells. ^18^F-FDG-PET has also been tested as a non-specific method to detect immune activation in a variety of infection and inflammatory diseases, which have been reviewed elsewhere (40, 41).

Though CT is also unable to specifically target a particular molecule or cell type, this modality has been evaluated for detection of response to TIL therapy (42). A faint halo was observed around melanoma lung metastases in 4 of 12 patients demonstrating partial response according to the Response Evaluation Criteria in Solid Tumors (RECIST). This halo is thought to occur due to perilesional hemorrhage. Neither pretreatment scans nor any of the 17 patients showing stable or progressive disease exhibited this effect.

## Direct Cell Labeling

For cancer therapy, ACT includes a variety of methods to collect, expand, and in some cases alter patient immune cells *ex vivo* prior to re-infusion. At least a proportion of TILs exhibit specificity for tumor antigen(s). Isolation, expansion, and re-infusion of these cells have been tested in various cancers including melanoma, head and neck squamous cell carcinoma, lung cancer, and genitourinary cancers (43). For patients who fail to generate endogenous anti-tumor immunity, T cells in the polyclonal blood pool can be engineered to express either a known tumor-specific T cell receptor or a synthetic MHC-independent CAR (43). Outside of the T cell compartment, expanded NK cells have also been evaluated for their therapeutic utility. ACT may benefit from imaging for non-invasive monitoring of survival, trafficking, and homing locations of transferred cells.

Direct radiolabeling of adoptive cells by passive incubation with radionuclide is a straightforward approach to track their fate *in vivo*. ^111^In is a popular radionuclide of choice which allows for imaging up to 96 h after injection, owing to its long half-life (Table 1). CD8^+^ cytotoxic T lymphocytes (CTLs) specific to the melanoma antigen Melan-A were ^111^In-labeled, infused in patients, and evaluated using serial whole body and static gamma camera imaging (44). Imaging was able to track CTL in sites of metastasis, as well as lung, liver, and spleen. A pre-clinical study also tested the efficacy of directly radiolabeling adoptive T cells with detection by SPECT imaging (3). T cells engineered to clonally express a T cell receptor (TCR) specific to hyaluronan (HA) were labeled with ^111^In and infused into BALB/c mice bearing both HA^+^ CT44 and HA^−^ CT26 tumors. Within 24 h post-injection, labeled CTLs could be detected in the CT44 tumor, with the concentration increasing over the duration of the study. Uptake in CT26 tumors remained low, suggesting TCR specificity was responsible for tumor homing. Human CAR-T cells have also been directly labeled with ^111^In for preclinical testing of migratory patterns in immunocompromised mice after different routes of injection, with intravenous showing optimal distribution vs. intraperitoneal or subcutaneous injection (45).

Multimodal imaging with concurrent SPECT/^18^F-FDG-PET is possible due to the differences in energies of the radionuclides used for each. A clinical study performed by Bernhard et al. expanded HER-2-specific T cells *ex vivo* and radiolabeled with ^111^In prior to reinfusion in a patient with HER2-overexpressing breast cancer (46). Accumulation of the cells was observed in bone marrow, where disseminated tumor cells were present and therapeutically eliminated. However, colocalization within solid tumors detected by ^18^F-FDG and/or MRI imaging was largely absent. Off-target homing of labeled cells was detected in lung, spleen, and non-tumor regions of the liver. This dual imaging approach was tested more recently in a single breast cancer patient (from clinical trial NCT00791037) with extensive bone-restricted metastases (47). Anti-HER2 T cells were ^111^In–labeled, with no evidence of impact on cell viability or function. After infusion, SPECT imaging revealed uptake of the tracer in various metastatic loci including the skull, sternum, and humerus within 24 h. Off-target tracer uptake was also observed in the spleen, liver, and heart. Concurrent ^18^F-FDG-PET showed increased signal in tumor sites through 48 h, suggesting potential detection of T cell metabolic activity.

**Figure 2:**
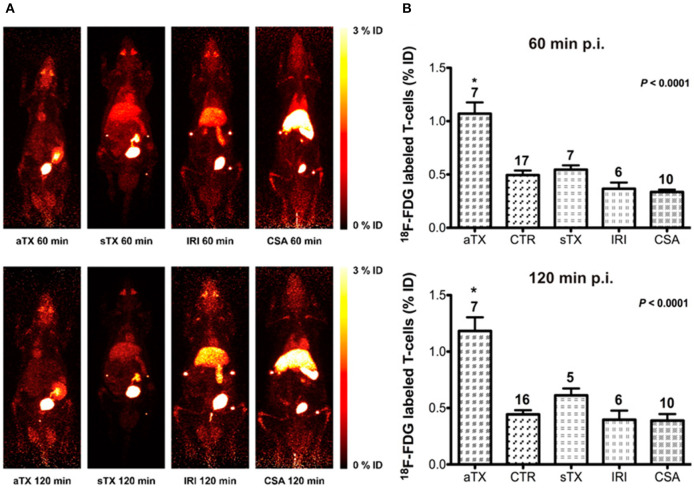
Direct cell labeling was utilized to examine acute rejection in rats with renal allografts (aTx) compared to control kidneys (CTR), syngeneic xenografts (sTx), and models of ischemia-reperfusion injury (IRI), and acute Cyclosporin A toxicity (CSA) by examining ^18^F-FDG-labeled T cells uptake. They identified significantly higher CD3 accumulation in the acute rejection model compared to the aforementioned models. **(A)** Maximum intensity projection (MIP) whole-body PET images of rats imaged with ^18^F-FDG-labeled T cells to examine renal allograft rejection. **(B)** The accumulation of the T lymphocytes present in the kidneys is expressed as percent injected dose ± standard error of the mean (%ID ± SEM). This research was originally published in Grabner et al. (48). Permission to reproduce this image has been obtained from the Journal of Nuclear Medicine.

^18^F labeled T cells with PET imaging has also been tested to monitor acute transplant rejection (48). The brown Norway-to-Lewis rat model is commonly used in transplantation studies because the dominant immunologic response is rejection. Allogenic human T cells were labeled with ^18^F-FDG *ex vivo* then injected into rats that had received renal transplants (Figure 2). They found tissue-specific detection of ^18^F accumulation in acute rejection mice compared to control naïve mice and mice with non-T cell-mediated acute tubular necrosis or acute cyclosporine A-induced nephrotoxicity. While the authors validated their findings with CD3 immunohistochemistry (IHC), a caveat to this approach for renal imaging is urinary excretion of the radioisotope. Additionally, the short half-life of ^18^F does not lend itself well to long-term *in vivo* monitoring after direct cell labeling.

PET-based cell trafficking has also been tested using ^89^Zr labeling of adoptively transferred cells (49–54). A study by Weist et al. imaged ^89^Zr-oxine-labeled human CAR-T cells with a labeling efficiency of 75% (50). This labeling method has also been tested in various murine models, but an efflux of ^89^Zr as well as chemotoxicity and radiotoxicity have been observed (51–54). To combat ^89^Zr-oxine efflux, Bansal et al. covalently labeled ^89^Zr to cell surface proteins and tested the cell trafficking of three different cell types: mouse derived melanoma cells, mouse dendritic cells, and human mesenchymal cells (49). In this study, the use of ^89^Zr allowed for the monitoring of the cell populations up to 7 days post-labeling, however they only achieved 30–50% labeling efficiency. No efflux was detected over the 7 days post-injection, nor was there evidence of chemotoxicity or radiotoxicity. It is important to note, *in vivo* detection of ^89^Zr has been reported as late as 30 days (~9 half-lives) after injection (55).

Aside from T cells, dendritic cell (DC) vaccines may benefit from imaging to track their localization *in vivo*. DC precursors are collected for expansion, maturation, and antigen loading *ex vivo* prior to re-infusion, where they process and present antigen to T cells, inducing effector and memory CD8^+^ and helper CD4^+^ T cells (56). DC vaccines have been tested against HIV-1 and various cancers (57–60). Efficacy of DC immunization is greatly influenced by migration to the lymph nodes, which may serve as an early biomarker for patient response (60). The ability to track DCs *in vivo* would thus aid in the success of treatment. DCs labeled with SPIONs have been detected in draining lymph nodes by MRI (61). Visualization of SPION-labeled DCs *in vivo* may be improved by increasing the intracellular nanoparticle concentration (e.g., by using higher concentrations during *in vitro* loading) or by enhancing the strength of the nanoparticle magnetic properties by surrounding its iron core with an oxide shell, Fe NP (62).

MRI detection of SPIONs has also been utilized to observe macrophage, T cell, and myeloid-derived suppressor cell (MDSC) trafficking. Due to their phagocytic characteristics, macrophages readily endocytose SPIONs *in vivo* and thus can be tracked by MRI imaging for a variety of indications, including inflammation, cancer, infection, and transplant rejection (31, 63, 64). Additional nanoparticle labeling methods for imaging macrophages are extensively reviewed by Weissleder et al. (65). T cell and MDSC labeling with SPION is more complex, requiring *ex vivo* manipulation, and thus this approach has so far been restricted to preclinical use (66–68). All pre-clinical tracers, including those discussed in direct cell labeling, are briefly summarized in Table 2; clinical tracers are discussed in Table 3.

**Table 2:** Pre-clinical studies of immune imaging methods and respective targets.

**Method**	**Cell type/target/tracer**	**Modality**	**Imaging agent**	**Target disease and study overview**
Direct Cell Labeling	Adoptive T cells	SPECT-CT	^111^In	Clonal expression of TCR specific to hyaluronan for CT26 (HA^+^) and CT44 (HA^−^) (3)
	CAR-T cells	SPECT-CT	^111^In	Breast cancer, human squamous cell carcinoma (45)
		PET	^89^Zr	Murine xenograft models of glioblastoma and subcutaneous prostate tumors (50)
	Allogenic human T cells	PET	^18^F-FDG	Renal transplanted rats (48)
	Macrophages	MRI	SPION	Assessment of cancer, inflammation, infection and transplant rejection (31, 63–65)
	DCs	PET	^89^Zr	^89^Zr-DBN labeling of mouse DCs (49)
		MRI	SPION	Track murine DCs trafficking to lymph nodes (61)
			Fe NP	DC based vaccination (62)
	MDSCs	MRI	SPION	MDSC biodistribution in murine hepatic colon cancer (68)
Reporter Gene Technology	FHBG	PET	^18^F	HSV1-tk reporter gene expression in glioma (69)
	hdCKDM	PET	^18^F-FEAU	dCKDM-transduced human PSMA CAR T cells in PSMA^+^ lung metastases of prostate cancer (70)
	hdCK3mut	PET	^18^F-L-FMAU	ACT monitoring (71)
	eDHFR	PET	^18^F-TMP	eDHFR-expressing anti-GD2 CAR-T cells in osteosarcoma and HCT116 colon cancer tumors (72)
	Human sodium iodide symporter (hNIS)	SPECT/CT	^99m^TcO4^−^	CAR-T cell therapy specificity and tracking (73)
				Treg tracking (74)
		PET/CT/BLI	^124^I and firefly luciferase	BMDC trafficking (75)
			^18^F-TFB and effluc gene	DC trafficking with murine DC2.4 (76)
	DC2.4/Effluc	BLI	effluc	Trafficking of murine DC2.4 co-expressing effluc and Thy1.1 (77)
	hNET	PET or SPECT	^124/123^I-MIBG ^124^I-FIAU	ACT monitoring vs. HSV-tk (78)
	SSTR2	PET	^68^Ga-DOTATOC	ACT monitoring to 8505c-FLuc^+^GFP^+^ thyroid tumors
	hNET, hNIS, hdCKDM, HSV1-TK	PET or SPECT/CT	^18^F-MFBG ^123^I-MIBG ^124^I-Iodide	Comparative study of reporter gene technology and T cell numbers (79)
	DAbR1	PET or SPECT	^86^Y/^177^Lu-AABD	CAR-T tracking with genetically introduced single chain fragment of 2D12.5/G54C (80)
	PSMA	PET	^18^F-DCFPyL	Anti-CD19 CAR-T cells transduced with PSMA for CAR-T cell monitoring (81)
Antibodies to immune cell populations	CD3	PET	^89^Zr	Syngeneic bladder cancer BBN975 (82)
				Syngeneic colon cancer with CTLA-4 therapy (83)
				Spontaneous mouse salivary and Neu^+^ mammary tumors (84)
	Visilizumab (CD3)	Scintigraphy	^99m^Tc	HuT78 human lymphoma, human peripheral blood mononuclear cells (85)
			[^99m^Tc]OKT3	Renal transplants (86)
		MRI		Acute collagen-induced arthritis in Lewis rats using IOPC (29)
		Ultrasound		Acute cardiac transplantation rejection in rats (28)
				Acute renal allograft rejection in rats (87)
	CD2	PET	^89^Zr	Pan T-cell imaging in ML2 leukemia tumors (88)
	CD7			
	CD8	PET	^64^Cu	Antigen-positive, antigen-negative, immunodeficient, antigen-blocked, and antigen-depleted mice (89)
			^89^Zr	Monitoring immunotherapy response in lymphoma and colorectal cancer (90)
	CD4	SPECT-CT	^111^In	Dextran sulfate sodium-induced colitis (34)
		PET	^89^Zr	Murine model of inflammatory bowel disease (91, 92)
	CD20	PET/CT	^89^Zr	Lymphoma xenografts (93)
			^64^Cu	Humanized transgenic mouse model of B-cell non-Hodgkins lymphoma (94)
			^89^Zr/^124^I	Malignant and normal B cells with obinutuzumab antibody fragments (95)
			^18^F	Human B cell lymphoma (96)
	CD19	PET	^64^Cu	Multiple sclerosis (97)
	CD11b	PET	^89^Zr	Murine colitis (98)
Antibodies to Surface Costimulatory orCorepressor Molecules	Anti-PD-L1	SPECT-CT	^111^In	NSG mice with CHO-PD-L1, triple negative breast cancer (MDA-231, SUM-149), NSCLC (H2444 and H1155) (99)
				Breast cancer MDA-MB-231, SK-Br-3, SUM149, BT474, MCF-7) (100)
				*neu*-N transgenic mouse model with NT2.5 and 4T1(101)
		Optical Imaging	NIR Licor800 dye	NSG mice with CHO-PD-L1, triple negative breast cancer (MDA-231, SUM-149), NSCLC (H2444 and H1155) (99)
		PET	^64^Cu	Breast cancer MDA-MB231, SUM-149, CHO-PD-L1 and CHO, syngeneic 4T1(102, 103)
		PET/CT	^18^F/^64^Cu	Brown adipose tissue (104)
			^89^Zr	LN229 glioblastoma (105)
	PD-1/PD-L1	PET	^64^Cu	Brown adipose tissue (106)
	Anti-mouse-PD-1	PET	^64^Cu	B16F10 melanoma (107)
				NSG/A375 melanoma (108)
	CTLA-4	PET	^64^Cu	CT26 colorectal tumors (109)
				NSCLC A549, H450 and H358 (110)
	OX40	PET	^64^Cu	A20 Lymphoma (111)
Antibodies to cytokines	IFN-γ	PET	^89^Zr	Mouse models of breast cancer (84)
	TGF-β	PET	^89^Zr	Mouse ovarian and human triple negative breast cancer (112)
	IL-1β	PET	^89^Zr	Murine colitis (98)
Directly labeled cytokines, ligands and peptides	IL-2	PET	^18^F	TC-1 mouse lung tumors subjected to radiation therapy alone or combination with HPV vaccine (113)
	IL-12	Scintigraphy	^99m^Tc	Imaging activated T lymphocytes and comparing biodistribution of mice with autoimmune colitis (114)
	14 kDa ectodomain fragment of PD-1	PET	^64^Cu	CT26 colorectal tumors (115)
	WL12	PET	^64^Cu	Various human and murine xenograft models (116–118)
			^68^Ga	
			^18^F	
	Granzyme B	PET	^68^Ga	CT26 colorectal tumors (119)

*TCR, T-cell receptor; HA, Hyaluronan; ^18^F-FHBG, 9-[4-[^18^F]fluoro-3-(hydroxymethyl)butyl]guanine; hdCKDM, deoxycytidine kinase double mutant; ^18^F-FEAU, 2′-[18F]fluoro-5-ethyl-1-beta-D-arabinofuranosyluracil; PSMA, prostate specific membrane antigen; hdCK3mut, deoxycytidine kinase triple mutant; ^18^F-L-FMAU, 2′-deoxy-2′-[^18^F]-fluoro-5-methyl-1-β-l-arabinofuranosyluracil; ACT, Adoptive cell therapies; TMP, Trimethoprim; eDHFR, E. coli dihydrofolate reductase; CAR, Chimeric Antigen Receptor; BMDC, Bone marrow derived DC; TFB, Tetrafluoroborate; hNET, human norepinephrine transporter; MIBG, metaiodobenzylguanidine; 124I-FIAU, [124I]2′-deoxy-2′-fluoro-β-d-arabinofuranosyl-5-iodouracil; SSTR2, somatostatin receptor 2; 68Ga-DOTATOC, gallium-68-chelated octreotide analogue tracer; MFBG, meta-fluorobenzylguanidine; DAbR1, DOTA antibody reporter 1; AABD, (S)-2-(4-acrylamidobenzyl)-DOTA; ^18^F-DCFPyL, Urea based PSMA targeting agent; FDG, Fluorodeoxyglucose; SPION, Superparamagnetic iron oxide nanoparticles; DCs, Dendritic Cells; ^89^Zr-DBN, ^89^Zr-desferrioxamine-NCS; Fe NP, Iron Nanoparticles; MDSCs, myeloid-derived suppressor cell; NSG, non-obese diabetic sever-combined immunodeficient gamma; NSCLC, non-small cell lung cancer; HPV, human papilloma virus; Treg, regulatory T cells*.

**Table 3:** List of clinical studies for immune imaging.

**Method**	**Cell type/target/tracer**	**Modality**	**Imaging agent**	**Target disease and study overview**
Direct Cell Labeling	TIL therapy	CT		Pulmonary metastases of metastatic melanoma (42)
	CTL	Static Gamma Camera	^111^In	Melanoma antigen Melan-A (44)
	HER-2-specific T Cells	SPECT/PET/CT	^111^In/^18^F-FDG	HER-2 overexpressing breast cancer (46, 47)
Reporter Gene Technology	FHBG	PET	^18^F	HSV1-tk reporter gene expression in Glioma (69, 120, 121)
Antibodies to immune cell populations	CD3	Scintigraphy	^99m^Tc	Rheumatoid arthritis, juvenile idiopathic arthritis, osteoarthritis & gouty arthritis (122)
			[^99m^Tc]OKT3	Rheumatoid arthritis synovitis (123)
				Rheumatoid arthritis and psoriatic arthritis for identification of inflamed synovium (124)
	CD20	PET/CT	^124^I	Rheumatoid Arthritis (125)
Antibodies to cytokines	TNF-α	Scintigraphy	^99m^Tc	Refractory sarcoidosis (126, 127)
Directly labeled cytokines, ligands and peptides	IL-2	Scintigraphy	^123^I/^99m^Tc	Crohn's, atherosclerosis and Type 1 diabetes (21–24, 128)
		SPECT/MRI	^99m^Tc	Diabetes detection of insulitis in autoimmune diabetes in adults (129)

*TIL, Tumor Infiltrating Lymphocyte; CTL, Cytotoxic T Lymphocyte; ^18^F-FHBG: 9-[4-[18F]fluoro-3-(hydroxymethyl)butyl]guanine; HSV1-tk, herpes simplex virus type 1 thymidine kinase*.

## Reporter Gene Technology

For ACT, reporter genes can be introduced *ex vivo* to allow for *in vivo* cell tracking. Reporter constructs encode proteins that either facilitate tracer uptake or directly modify tracer constructs to promote sequestration specifically in adoptive cells. Ideally, the result should be passive and allow flexibility in the timing of imaging without disruption of normal function or introduction of immunogenicity. Another advantage is the option for longitudinal monitoring of ACT over more than one time point. Compared to direct cell labeling, this approach permits monitoring at multiple time points after therapeutic cell delivery. A variety of reporter genes have been tested for ACT applications, including herpes simplex virus type 1 thymidine kinase (HSV1-tk), deoxycytidine kinase double and triple mutants (hdCKDM and hdCK3mut, respectively), *E. coli* dihydrofolate reductase (eDHFR), sodium-iodide symporter (NIS), norepinephrine transporter (NET), and somatostatin receptor 2 (SSTR2) (69–73, 75–79, 120, 121, 130). To date, no indication of reduced ACT function has been described for any of these technologies.

### Enzymatic Reporter Genes

Enzymatic substrate modification is a practical approach that yields specific tracer retention in adoptive cells. To date, several enzyme/substrate combinations have been evaluated. The HSV1-tk enzyme has been paired with pyrimidine nucleoside or acycloguanosine analog tracers that are unmodified by mammalian thymidine kinases, limiting tracer accumulation to reporter gene-transduced adoptive cells. Clinical application of this technology is reported for HSV1-tk-transduced CAR-T cells engineered to express an interleukin-13 (IL-13) zetakine, which targets IL-13 receptor alpha 2-expressing glioblastoma (69, 120, 121). Reporter gene engineered cells are detected by 9-[4-[^18^F]Fluoro-3-(hydroxymethyl)butyl]guanine ([^18^F]FHBG), a high-affinity substrate with an investigational new drug designation (IND #61,880). Normal routes of clearance of [^18^F]FHBG are hepatobiliary and kidney. An increase in tracer uptake was detected by PET imaging 1-week post-ACT compared to pre-treatment scans. Some variability in pretreatment uptake was noted between patients, which was attributed to disruption of the blood-brain barrier (121). These findings highlight the need for comparative scans before and after treatment to assess changes in uptake rather than a single post-treatment scan.

A major caveat to the HSV1-tk reporter gene is the high likelihood of pre-existing CD8 T cell immunity to HSV, which could mediate elimination of adoptive cells. Additionally, certain chemotherapy and bone marrow transplant regimens utilize prophylactic antiviral ganciclovir, a prodrug that kills HSV-tk-expressing cells. To reduce immunogenicity and prevent antiviral drug toxicity, various mutants of the human deoxycytidine kinase (dCK) have been generated with amino acid substitutions in the active site, which promotes selective phosphorylation of fluorinated (to permit ^18^F incorporation) thymidine analogs. The double mutant reporter gene dCKDM tested by Likar et al. shows ~100-fold increased tracer substrate uptake and retention compared to native dCK, with reduced sensitivity to acycloguanosine-derived drugs (70). dCKDM-transduced human prostate specific membrane antigen (PSMA)-specific CAR-T cells were detected in PSMA-expressing prostate metastases to the lungs with 2′-[^18^F]fluoro-5-ethyl-1-beta-D-arabinofuranosyluracil ([^18^F]-FEAU) and PET imaging the same day as adoptive cell delivery. Another construct encoding the triple mutant hdCK3mut was co-expressed with a melanoma antigen-specific T cell receptor and tested with PET reporter 2′-deoxy-2′-[^18^F]-fluoro-5-methyl-1-β-l-arabinofuranosyluracil ([^18^F]-L-FMAU) (71). Tracer signal was comparatively higher in tumors with matching HLA vs. contralateral HLA-mismatched tumors, supporting the utility of monitoring ACT with hdCK3mut. Importantly, engineered cells demonstrated no changes in function or viability from expression of these reporter genes.

A reporter construct encoding the *E. coli* dihydrofolate reductase (eDHFR) enzyme facilitates specific retention of labeled antibiotic compound trimethoprim (TMP). The tracer [^18^F]fluoropropyl-trimethoprim ([^18^F]-TMP) has been tested in a preclinical model of eDHFR-expressing human anti-GD2 CAR-T cells (72). Localization of [^18^F]-TMP increased 6- to 8-fold between days 7 and 13 after ACT in GD2-expressing 143b osteosarcoma tumors, compared to under 4-fold in GD2-deficient HCT116 colon cancer tumors. Focal intratumoral tracer uptake was validated by IHC for human CD8. Because mice also exhibited non-specific bone and gut uptake, a [^18^F]-TMP biodistribution assay was also conducted in non-human primates. Liver and kidney uptake (routes of excretion) were predominant with limited bone or gut involvement, supporting clinical translation of [^18^F]-TMP.

### Importer Reporter Genes

Selective tracer import is another common approach for ACT imaging. Examples of transduced genes include the sodium iodide symporter (hNIS), human norepinephrine transporter (hNET), and somatostatin receptor 2 (SSTR2). hNIS has been tested with a variety of cell types, including CAR-T cells (73), regulatory T cells (Tregs) (74), and DCs (75, 76).

Emami-Shahri et al. performed a pre-clinical study using the PSMA-specific P28ζ CAR-T cell engineered to co-express hNIS in mice bearing PSMA-expressing tumors (73). SPECT was used to detect hNIS-mediated technetium-99m pertechnetate (^99m^${\text{TcO}}_{4}^{-}$) uptake. Imaging was conducted weekly for long-term monitoring of transferred cells up to 21 days after delivery, and intratumoral T cell infiltration was validated *ex vivo* by IHC at the termination of the study. The authors observed a correlation between therapeutic response and focal intratumoral uptake as early as 9 days post-treatment. A potential drawback was the off-target uptake in the stomach and thyroid, which, paired with the normal route of clearance through the kidneys and bladder may obscure CAR-T cell specific uptake in any of these regions.

**Figure 3:**
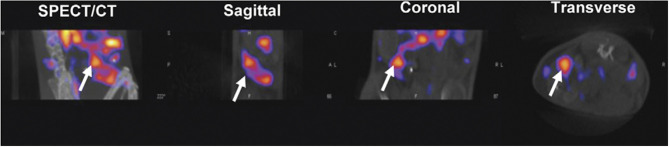
Reporter gene technology. Tregs were transduced with a reporter gene for the Sodium Iodide Symporter (NIS) for specific ^99m^${\text{TcO}}_{4}^{-}$ uptake. C57BL/6 mice were injected with NIS Tregs and injected 1 day later with ^99m^${\text{TcO}}_{4}^{-}$ to examine Treg uptake in the spleen, demonstrating *in vivo* radiolabeling of Tregs. Mice were imaged by NanoSPECT/CT with a focus on the spleen (white arrow). This research was originally published in Sharif-Paghaleh et al. (74). Permission to reproduce this image has been obtained from the PLOS One.

Treg imaging may be useful for monitoring suppressive immune cell localization after organ transplant or during autoimmune disease onset or treatment. A preclinical study transduced *ex vivo*-expanded, autologous Tregs with the hNIS reporter, with detection by ^99m^${\text{TcO}}_{4}^{-}$ via SPECT/CT imaging (Figure 3) (74). SPECT imaging showed higher accumulation of signal within the spleen in comparison to other organs. It has yet to be determined if this technology can be utilized in circumstances of inflammation or autoimmunity.

Ahn et al. used a multimodal reporter system of bioluminescence imaging (BLI) and PET to monitor bone marrow derived DC (BMDC) trafficking by BLI and PET imaging with ^124^I (75). Mice were pre-treated with Tumor Necrosis Factor-α (TNF-α) 24 h prior to DC delivery as a means of enhancing migration to the draining lymph node. On day 7 post-foot pad injection, both BLI and ^124^I PET showed increased draining node signal. The authors showed reporter gene-transduced DCs remained effective as a prophylactic tumor lysate vaccine, suggesting retention of function. Another report used the same combined luciferase/hNIS reporter for imaging the murine DC line DC2.4 (76). Cells were injected either intramuscularly or in the foot pad and were imaged using BLI and a novel hNIS transport-specific PET probe, ^18^F-tetrafluoroborate (^18^F-TFB). Signal enhancement in the draining popliteal lymph node over time was suggestive of DC trafficking. For intramuscular delivery BLI outperformed PET for early 1-day p.i. imaging due to elevated non-specific background of ^18^F-TFB, which cleared within 4 days p.i. Tracer signal of reporter gene-transduced DCs by ^18^F-TFB was less intense than BLI after 2 days p.i., however both the injection site and draining nodes were detectable. More recently, this same group utilized BLI alone for *in vivo* tracking of DC localization (77).

hNET is another non-immunogenic reporter gene option which mediates specific uptake of clinical grade metaiodobenzylguanidine (MIBG) or meta-fluorobenzylguanidine (MFBG). As a direct comparison of hNET vs. HSV1-tk for ACT monitoring, GFP-transduced EBV-specific T cells were separated into hNET-transduced CD4^+^ and HS1V-tk-transduced CD8^+^ subsets. These cells were injected intratumorally into EBV^+^ tumor xenografts and imaged after injection of radiotracer ^124^I-MIBG (hNET) or ^124^I-FIAU (HSV1-tk) (78). Signal intensities in the tumor closely correlated to the number of T cells injected for both subsets. Prolonged serial PET imaging showed T cells increased in the targeted tumor by more than 12-fold over 28 days of observation, potentially due to intratumoral T cell proliferation. SPECT imaging with ^123^I-MIBG also produced a signal intensity that closely correlated with the dose of T cells delivered, suggesting either imaging modality may be used.

SSTR2 is also non-immunogenic and has been tested for monitoring CAR-T cells in a preclinical model using a clinically approved gallium-68–chelated octreotide analog tracer (^68^Ga-DOTATOC) with PET imaging in lung-localized 8505C tumors (130). While this approach was able to monitor adoptive T cells *in vivo*, it was noted that non-specific accumulation of ^68^Ga-DOTATOC in the thoracic cavity increased in parallel with tumor burden, suggesting this tracer may be problematic in circumstances of leaky vasculature. However, intratumoral ^68^Ga-DOTATOC uptake matched tumor growth patterns, where PET signal initially increased followed by a decrease in resolving tumors. Validation by IHC showed correlation between tracer uptake and percent of CD3 infiltration.

A multimodal approach was used by Moroz et al. to directly compare sensitivity of hNET, hNIS, hdCKDM, and HSV-tk by injecting mice subcutaneously with a titration of reporter-transduced T cells followed by detection with various probes for PET or SPECT imaging (79). SPECT imaging was only marginally effective compared to PET, requiring >10^7^ T cells to produce a detectable signal with hNET and ^123^I-MIBG. Among the PET reporter/probe combinations tested, hNET paired with ^18^F-MFBG showed highest sensitivity, with detection of as few as ~40,000 injected cells. Overall, ^18^F-tagged probes outperformed ^124^I, with a trend toward higher sensitivity from probes exhibiting faster clearance.

### Genetically Introduced Cell Tags

Similar to the standard reporter genes described above, efforts to engineer adoptive cells to express a tracer-detectable surface marker are also underway. The DOTA antibody reporter 1 (DAbR1), consisting of a single-chain fragment of anti-Y-DOTA antibody 2D12.5/G54C fused to the human T-cell transmembrane domain, can be detected by covalent binding of radiolabeled lanthanoid (S)-2-(4-acrylamidobenzyl)-DOTA (AABD) (80). DOTA has been utilized for multiple radiopharmaceutical tracers and contrast agents. AABD was labeled with ^86^Y or ^177^Lu for PET or SPECT imaging, respectively. Cells were detected as late as 24 h after tracer injection, while unbound tracer continued to clear over time. Autoradiography and IHC staining of CD3 confirmed radiotracer activity was highest in areas containing infiltrated CAR-DAbR1 T cells.

The self antigen PSMA was also tested as a potentially non-immunogenic target for PET imaging adoptive T cells using an ^18^F radiolabeled urea-based PSMA targeting agent, DCFPyL (81). T cells were transduced with a dual expression construct encoding a CD19 CAR and a modified PSMA molecule tPSMA^(N9del)^, which was designed to retain surface expression. T cell function was not impaired by PSMA expression, and the tracer was able to identify CAR T cell infiltration within primary and metastatic tumor sites. IHC was used to confirm T cell infiltrates in tissues with tracer uptake. Of course, this approach is best suited for imaging ACT against tumors lacking intrinsic PSMA expression.

## Antibody-Based Tracers

Monoclonal antibodies (mAb) are advantageous as tracers because of their high specificity, low immunogenicity if the Fc region matches host, and relative ease of labeling with a tracer. The various labeling approaches are reviewed (131). However, when using mAbs to target a specific cell population, it is important to evaluate the potential impact on cell viability, proliferation, and function *in vivo*. Certain clones, particularly when the Fc region is intact, have been shown to deplete their target cell population, reducing their applicability for imaging (82). To circumvent this, cleaved or engineered antibody fragments lacking Fc receptor can be designed. Antibody fragments also have the benefit of a faster route of clearance due to their smaller size, making them more amenable to imaging at earlier time points after tracer injection. The characteristics of intact, cleaved, and engineered antibodies, including serum half-life and clearance route, have been described in detail by Freise and Wu (131). Antibodies to surface receptors may also exhibit agonist or antagonist activities, which may disrupt cell function (90, 132).

When using mAb tracers to immune cell surface molecules, it is important to note pharmacokinetics, as target immune cell populations naturally accumulate in primary and secondary immune organs, such as the spleen and lymph nodes. These reservoirs absorb large amounts of tracer, a phenomenon often referred to as an antigen sink. The technique of pre-dosing injections or blocking with non-radiolabeled antibody prior to injection of the tracer can potentially decrease the antigen sink effect, however there is a fine balance between blocking the antigen sink and displacing target tissue uptake of the tracer (89). Another caveat to using an antibody as a carrier is the route of clearance. In general, the route of clearance is dictated by the size and/or charge of the molecule, in addition to presence of the Fc region. Full-length antibodies, or large antibodies fragments (>60 kDa) are cleared through the liver, whereas those >60 kDa are more rapidly cleared through the kidneys (133). Non-specific binding of radioactivity can be visible in organs such as the liver, kidneys, gallbladder, and the gastrointestinal tract (91).

### Antibodies to Immune Cell Populations

Antibody tracers to several cell classification proteins are reported in the literature to monitor the location, movement, and relative density of these cell populations *in vivo*. Examples are T cell markers CD3, CD4, CD8, CD2, and CD7; B cell marker CD20; and myeloid marker CD11b. CD3, found on all T cells, can be targeted to observe general T cell trafficking. CD3 mAb has been used with PET, scintigraphy, MRI and ultrasound imaging (28, 29, 82–87, 122–124). For PET imaging, CD3 has been used pre-clinically to observe T cell trafficking in bladder and mammary cancer, in addition to monitoring response to CTLA-4 therapy in colon cancer models (82–84). In these studies, full-length mouse mAbs were conjugated with ^89^Zr. The tracers were observed to accumulate in secondary lymphoid tissues including spleen, thymus, and lymph nodes, which is not unexpected due to the relative densities of CD3^+^ T cells in these organs. Each of these studies noted tracer uptake in the liver, which is a well-known route of full-length mAb clearance (27, 83, 134, 135). In Beckford's study, low uptake of the tracer was observed in bone, where free ^89^Zr accumulates (27, 135, 136). Given its proximity to the T cell receptor, antibodies to CD3 (e.g., mouse CD3 mAb clone 2C11) may induce expansion of T cells or affect T cell function. Upon *ex vivo* analysis, one study reported a relative depletion of CD4 T cells and an expansion of CD8 T cells after delivery of an ^89^Zr-anti-CD3 tracer (clone 17A2) in mice (82). Though tracers are generally dosed at sub-therapeutic levels, it is important to consider any potential downstream effects on target cells resulting from engagement with the tracer.

Scintigraphy imaging with technetium-99m (^99m^Tc) conjugated anti-CD3 has been utilized in humans and preclinical mouse models to monitor sites of inflammation, particularly for rheumatoid arthritis and transplant rejection (85, 86, 122–124). The majority of these were human studies utilizing OKT3, the first FDA approved therapeutic mAb, which is known to initially activate T cells with subsequent induction of apoptosis. The humanized mAb visilizumab, which binds selectively to activated T cells, was also tested for specific identification of human T cells in an immunocompromised mouse model (85). Though scintigraphy yields lower-resolution compared to PET or SPECT, each of the clinical studies clearly demonstrated uptake of the tracer in areas of T cell accumulation.

Non-radioactive alternatives have also been tested with CD3-specific tracers. One approach is MRI using SPION modified by carboxylation of the polyethylene glycol (PEG) coating surface, termed IOPC, which has been conjugated to a full-length anti-CD3 mAb (29). This tracer was able to monitor T cell infiltration during acute collagen-induced arthritis in Lewis rats. Ultrasound imaging with CD3 mAb-conjugated nanobubbles has also been evaluated in models of acute graft rejection (28, 87). Grabner et al. tested this system on renal transplants and were able to diagnose organ rejection with high specificity, differentiating from the non-T-cell-mediated pathologies acute tubular necrosis and calcineurin inhibitor toxicity. Jinfeng et al. used a similar approach, focusing on acute rejection following cardiac transplantation (28). Imaging revealed a rapid visual enhancement of the affected myocardium, where T cell infiltration was induced.

Aside from CD3, tracers to CD2 and CD7 were also designed to monitor the T cell population (88). CD2 and CD7 are both expressed on T and NK cells, and thus imaging agents against these markers will not be able to distinguish these two cell types. However, both T and NK cells can exhibit anti-tumor cytotoxicity, supporting the utility of these targets for imaging response to cancer immunotherapy. Mayer et al. tested the utility of mouse-anti-human antibody tracers against CD2 (clone OKT11) and CD7 (clone T3-3A1). Unlike a control anti-CD3 (clone OKT3) tracer, neither the CD2 nor the CD7 mAb or their respective F(ab′)_2_ fragments affected T cell proliferation or induction of apoptosis *in vitro*, with a slight increase in IFN-γ production from anti-tumor T cells exposed to anti-CD2. F(ab′)_2_ tracers were able to positively identify the accumulation of adoptively transferred anti-tumor T cells in subcutaneous human acute leukemia ML2 tumors in NSG mice. However, the anti-CD2 tracer resulted in systemic T cell depletion and lack of anti-tumor activity, even without an intact Fc region, highlighting the impact antibodies and their fragments may have on cell surface receptors.

**Figure 4:**
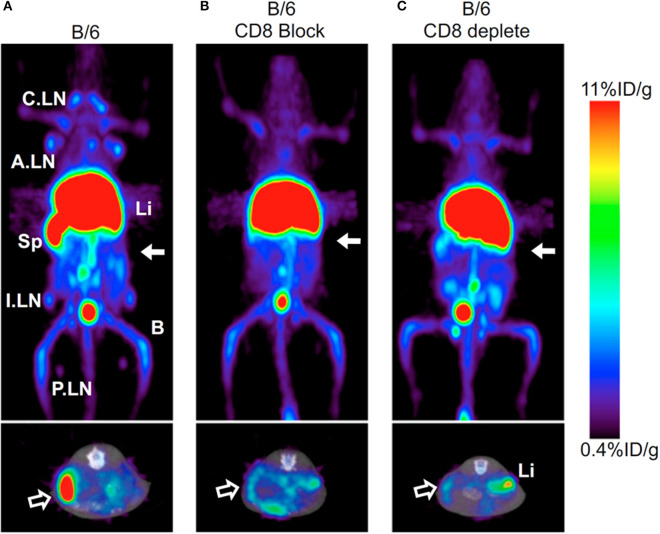
Antibody-based tracers. Antibody based tracers have been developed to target immune cell populations. However, one hurdle to overcome for some antibodies is Fc-mediated depletion. Tavaré et al. developed two ^64^Cu-NOTA anti-murine CD8 minibodies. **(A)** ^64^Cu-NOTA-2.43Mb exhibited targeted spleen uptake in B/6 mice. **(B,C)** Both the blocking cohort and CD8 depleted cohort displayed decreased spleen uptake (Upper images—Coronal MIPs, Lower images—Transverse). This research was originally published in Tavaré et al. (89). Permission to reproduce this image has been obtained from the Proceedings of the National Academy of Sciences of the United States of America.

Imaging the total T cell population may not be optimal given the diversity of populations within this subset. CD3, CD2, and CD7 imaging cannot discern whether detected cells are helper CD4, cytotoxic CD8, or regulatory T cells, which each play different functional roles in disease. A logical approach to fine-tune T cell imaging is to design probes specific for the two major subsets, CD4 and CD8. Currently CD8 T cells have been imaged using PET (89, 90). To avoid Fc-mediated depletion, which is common among CD8 mAbs, CD8 antibody fragments have been generated (Figure 4) (89). Minibodies lacking the full Fc domain (scFv-CH3) were engineered from CD8 mAb clones YTS169.4.2.1 and 2.43, labeled with ^64^Cu, and tested for uptake in lymphoid organs in mice under various conditions to determine specificity. A cys-diabody was also created using the variable regions of CD8 mAb YTS169.4.2.1 conjugated to ^89^Zr, which showed specificity and tracked CD8^+^ T cells in response to three different murine models of tumor immunotherapy: adoptive transfer of antigen-specific T cells, antagonist antibody, and checkpoint blockade (90). Both minibody and diabody tracers demonstrated faster specific uptake and clearance from background tissues compared to most reported full-length mAb tracers, supporting their use for shorter-lived radionuclides and same-day imaging.

**Figure 5:**
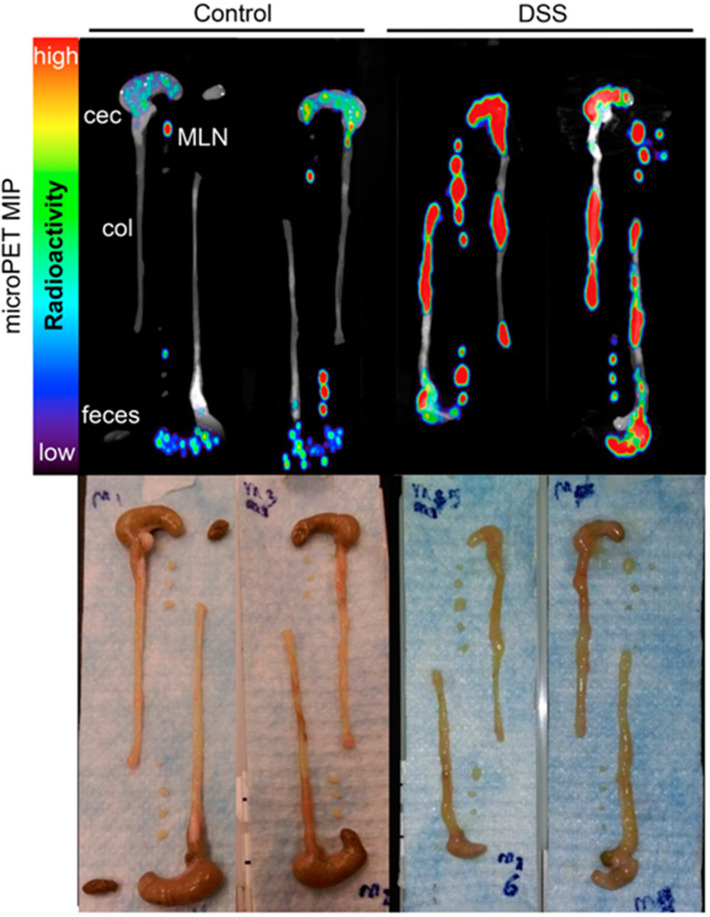
Antibody based tracers in non-cancerous diseases. ImmunoPET tracer development has also extended to immunogenic diseases such as Colitis. Freise et. al. imaged CD4^+^ T cells with ^89^Zr-malDFO-Gk1.5 cys-Diabody (cDb), to non-invasively monitor inflammation of the intestines caused by specific cell subsets. *Ex vivo* representative images of the colons, ceca and mesenteric lymph nodes (MLNs) are shown comparing tracer uptake in a dextran sulfate sodium (DSS) induced colitis model compared to control. The DSS mice had 3.1-, 3.9-, and 3.0-fold increased uptake in the colons, ceca, and MLNs, respectively. This research was originally published in Freise et al. (91). Permission to reproduce this image has been obtained from the Journal of Nuclear Medicine.

CD4^+^ T cell imaging may be of clinical value for immune-driven pathologies such as inflammatory bowel disease and multiple sclerosis (137, 138). PET and SPECT imaging have both been tested for monitoring CD4 T cells *in vivo* (34, 91, 92). A full length anti-CD4 antibody labeled with ^111^In was used for SPECT imaging in mice orally pre-treated with dextran sulfate sodium (DSS) which activates CD4^+^ T cells in the periphery and drives their influx into the colon, inducing colitis (34). SPECT imaging showed activity around the bowel, localized to areas of colonic inflammation that correlated with the degree of pathology. Freise et al. developed an anti-CD4 cys-diabody (cDb) tracer derived of mAb GK1.5 conjugated to ^89^Zr for PET imaging (91, 92). They found a dose-dependent effect of this tracer on CD4 T cells, where 40 μg of unlabeled cDb resulted in a transient decrease in CD4^+^ T cells within the blood, spleen, and lymph nodes, which did not occur when 2 μg was tested. As this tracer lacks an Fc region, these results suggest engagement of the CD4 receptor with this cDb clone functionally impacts T cells (92). Using the 2 μg dose of ^89^Zr-cDb, CD4 T cells were monitored in a mouse model of inflammatory bowel disease (Figure 5) (91). Increased signal in the mesenteric lymph nodes and colon was detected after induced inflammation compared to the untreated control mice, which was confirmed by CD4 IHC.

Development of B cell-specific tracers is also underway. Clinical trials using therapeutic anti-CD20 mAbs have been implemented to treat B cell lymphoma and autoimmune diseases including systemic lupus erythematosus, rheumatoid arthritis, and multiple sclerosis (139). CD20 is not degraded or internalized after antibody binding, supporting its use as an imaging target (140). Thus far, PET has been the most reported modality for antibody-based imaging of CD20^+^ cells (93–96, 125, 141). In addition to CD20, CD19 has also been a target for imaging B cells in multiple sclerosis by PET (97).

Several therapeutic anti-CD20 mAbs have been tested for imaging applications. A clinical study for rheumatoid arthritis used full-length rituximab labeled with ^124^I for PET/CT imaging (125). Uptake in affected joints was observed after 24 h in four of five patients, with detection of tracer accumulation also observed in joints that were not considered clinically inflamed. ^89^Zr-labeled rituximab was also used as a metric to predict the dose of radiation that patients would receive with ^90^Y-labeled rituximab with or without preloading of an unlabeled blocking mAb (141). Preclinical studies have been conducted to directly compare different full length CD20 antibodies for PET imaging: humanized obinutuzumab, fully human ofatumumab, chimeric antibody rituximab, and fully murine antibody tositumomab (93). All mAbs were labeled with ^89^Zr and used to detect human lymphoma xenografts. Imaging with mAbs containing human Fc showed similar or better tumor localization compared to murine Fc-containing mAbs, which supports their use clinically. Non-human Fc regions may generate an anti-idiotype antibody, hindering repeated injections.

In a study by Natarajan and colleagues, rituximab was compared to an engineered, low molecular weight protein scaffold that targets CD20, human fibronectin type 3 domain (FN3), for visualization and monitoring of human non-Hodgkin's lymphoma (94). This protein is engineered for affinity to human CD20 and contains 3 solvent-exposed loops which are structurally similar to the complementarity determining regions of an immunoglobulin (FN3-CD20). The smaller size of FN3 (~100 amino acids, or ~8% of a mAb) promotes rapid clearance while maintaining *in vivo* specificity. When rituximab and FN3-CD20 were labeled with ^64^Cu, FN3-CD20 had a higher spleen-to-blood ratio (the spleen is rich in CD20^+^ B cells) and allowed for better resolution at earlier time points. Both liver and kidneys were observed as clearance organs for this tracer.

Engineered anti-CD20 cDb and cys-minibody (cMb) fragments, generated based on the antigen recognition sequences of rituximab and obinutuzumab (GA101), have been tested with ^89^Zr vs. ^124^I labeling for PET imaging (95). Not surprisingly, the cDb and cMb showed higher tumor-to-blood ratios at an earlier time point vs. full-length mAbs. Interestingly, GA101 cDb and cMb outperformed their rituximab-based counterparts, suggesting these humanized antibody fragments may be better clinical candidates for imaging. ^89^Zr labeling also improved tumor signal over ^124^I, likely due to the residualizing characteristics of ^89^Zr. Internalization of the radiotracer was observed with the ^89^Zr labeled antibody fragments in naïve B cells, which was largely absent in B cell lymphoma cells.

Shorter-lived ^18^F has also been tested with a CD20 cDb in a mouse model of human B cell lymphoma using two different conjugation methods, N-succinimidyl 4-[^18^F]-fluorobenzoate (^18^F-SFB) and site-specific N-[2-(4-[^18^F]-fluorobenzamido)ethyl]maleimide (^18^F-FBEM) (96). Although FBEM conjugation had faster target tissue uptake and blood clearance, its hepatobiliary clearance was less favorable than that of SFB, which clears through the kidneys. Compared to the clinical gold-standard ^18^F-FDG, CD20 imaging was able to assess whole-body distribution of B cells regardless of metabolic activity level.

Anti-CD20 as well as anti-CD19 mAbs have been used therapeutically in multiple sclerosis patients (142). Stevens et al. developed an anti-CD19 tracer radiolabeled with ^64^Cu for preclinical use (97). Mice were induced with experimental autoimmune encephalomyelitis (EAE) and signs of paralysis were observed prior to whole-body PET imaging. Imaging revealed B cell infiltration to the central nervous system with significantly higher uptake in the brain of EAE mice compared to healthy controls. Gamma counts of the spinal cord *ex vivo* also indicated higher tracer uptake in the spinal cord of EAE mice compared to controls.

A tracer to CD11b has also been tested preclinically to image innate immune cells during chronic inflammation associated with inflammatory bowel disease (IBD) (98). During IBD, CD11b-expressing myeloid cells accumulate in the colon. An antibody to CD11b was radiolabeled with ^89^Zr for PET imaging, and colon uptake in IBD-induced mice was similar to ^18^F-FDG PET, with higher sensitivity than MRI. Biodistribution showed elevated ^89^Zr-anti-CD11b uptake in the gastrointestinal tract, spleen, liver, and bone marrow in colitis-induced mice compared to control, untreated mice. Spleen and bone marrow uptake are not surprising given the presence of myeloid and other CD11b^+^ cells in these tissues.

### Antibodies to Surface Costimulatory/Corepressor Molecules

With the clinical success of checkpoint blockade therapies, development of imaging agents for co-stimulatory and repressor molecules has gained traction, particularly for markers such as PD-1, PD-L1, and CTLA-4 (99–102, 104–108, 110, 115, 143). Alternatively, antibodies to activate the immune response through costimulatory receptors are also under investigation. Because the majority of patients are non-responsive to these therapies, there is a need to develop methods to predict the likelihood of patient response (111). In recent years, there has been a surge in checkpoint and costimulatory molecule imaging tracers for PET, SPECT, and optical imaging (OI) modalities.

The humanized antibody atezolizumab (anti-PD-L1) has been utilized as a tracer candidate in all three (PET, SPECT, and OI) modalities. Chatterjee et al. developed both ^111^In SPECT and near-infrared (NIR) Licor800 dye-labeled anti-PD-L1 tracers, both of which delineated differential PD-L1 expression in transfected CHO cells and several orthotopic tumor xenografts (99). The authors suggested NIR-PD-L1 tracers may have bronchoscopic or thorascopic application for detection of PD-L1 expression in lung tumors. Another anti-human-PD-L1 antibody (clone EPR19759) was tested for NIR imaging of subcutaneous tumors from SW620, SW480, and HCT8 human colorectal cancer lines in nude mice (143). Tracer uptake was proportional to the PD-L1 expression levels of these tumor cells. More recently, atezolizumab has also been developed as a PET tracer using ^64^Cu (102, 103). Since atezolizumab has cross-reactivity to human and mouse, this tracer has been tested in both human xenografts and immunocompetent BALB/c mice with syngeneic mammary carcinoma 4T1. PD-L1 tracer uptake was observed in the brown adipose tissue (BAT) of immune-compromised vs. immunocompetent mice, showing higher localization within the BAT in the latter cohort. Other groups have also noted that BAT has endogenous PD-L1 expression and it is visualized when imaging with PD-L1 tracers (104, 106). Additional PD-L1 antibody clones have also been explored, such as the murine mAb PD-L1 3.1, which was also tested with ^111^In labeling and SPECT imaging, again demonstrating capacity to detect various PD-L1 expression levels on tumors. This same PD-L1 3.1 clone labeled with ^111^In for SPECT imaging was also tested using the *neu*-N transgenic mouse model with subcutaneous isografts of the NT2.5 cell line (101). Tracer uptake was detected in tumor, spleen, liver, thymus, heart, and lungs in this immunocompetent model.

Engineered antibody fragments have also been developed as tracers to detect PD-L1. Ingram et al. developed camelid single-domain antibodies, or VHHs, that target PD-L1, which were used for specific BAT detection in both BALB/c and C57BL/6 mice (104). KN035 is a 79.6 kDa engineered human anti-PD-L1 comprised of an Fc tail with two single chain domains, and is an attractive option for imaging because of its IND designation for both advanced and metastatic solid tumors (105). Biodistribution studies were conducted using ^89^Zr in both tumor xenograft-bearing mice and tumor-naïve healthy non-human primates (NHPs). Although liver and gallbladder uptake were observed in the NHP study, thought to be a main mechanism of clearance, high renal uptake was also observed, likely due to a c-terminal amino acid sequence of KN035 that interacted with kidney proximal tubuli. Although antibody fragments are typically best paired with shorter-lived radionuclides, the high liver uptake throughout the study suggested the need for a longer-lived radionuclide to reduce the background ratio.

Preclinical studies have been conducted to test several PD-1-specific tracers, with the goal of monitoring expression on TILs. A ^64^Cu-radiolabeled anti-mouse PD-1 antibody (clone J43) was tested in a B16F10 melanoma model, and uptake was detected in tumor and spleen (107). Additionally, the humanized antibody pembrolizumab has also been radiolabeled with ^64^Cu for PET imaging (108). Humanized mouse models were used to observe antibody specificity to human PD-1 without interfering cross-reactivity to murine PD-1. PET imaging in the NSG/A375 melanoma model showed ^64^Cu-pembrolizumab bound to human PD-1 expressed on a subpopulation of TILs.

PET tracers to CTLA-4 are also in development to identify potential responders to checkpoint blockade treatment. Conjugated to ^64^Cu, a full-length mouse CTLA-4 antibody (clone 63828) was tested in CT26-bearing immune competent BALB/c mice (109). PET images showed significant accumulation in the tumor compared to the control tracer with the specificity to CTLA-4 being confirmed by *ex vivo* studies. Another study used human-derived A549, H460, and H358 NSCLC cell lines in immunocompromised mice, which have varying levels of intrinsic CTLA-4 expression (110). Ipilimumab conjugated with ^64^Cu showed high accumulation in A549 tumors, with lower uptake in H460 and H358, demonstrating the quantitative capacity of PET imaging.

Co-stimulatory molecules such as OX40 have also been examined as a means of imaging activated T cells. Compared to CD3, which is on the surface of all T cells, OX40 is induced upon T cell activation, and is thus representative of T cell behavior. Alam et al. developed an OX40 mAb for PET imaging by conjugation to ^64^Cu (111). The tracer was able to discern bilateral tumors where only one was treated with immune-stimulating CpG, suggesting the potential to characterize immunologic activity *in situ*.

### Antibodies to Cytokines

Our group recently reported targeting of IFN-γ to image response to tumor immunotherapy (84). IFN-γ PET, using ^89^Zr-conjugated anti-IFN-γ mAb AN-18, was examined in mouse models of breast cancer in response to a HER2 cancer vaccine. Vaccinated mice imaged with ^89^Zr-anti-IFN-γ exhibited significantly higher tumor uptake than control, untreated mice. Importantly, there was no significant change in uptake of a non-specific IgG tracer between the control and vaccinated groups, suggesting ^89^Zr-anti-IFN-γ uptake was indicative of vaccine response and not an artifact of enhanced permeability and retention (EPR) in the tumor. Of note, in an immune tolerant spontaneous tumor model, IFN-γ PET demonstrated better sensitivity to detect anti-tumor immunity than peripheral T cell analysis, suggesting *in situ* immune evaluation may serve as a better indicator of response.

TNF-α is a proinflammatory cytokine that has been extensively targeted clinically with blocking antibodies for rheumatoid arthritis, inflammatory bowel disease, and sarcoidosis (144). Two clinical studies report using ^99m^Tc radiolabeled TNF-α mAb infliximab for scintigraphy imaging to both monitor refractory sarcoidosis disease activity and evaluate potential patient response to anti-TNF-α treatment (126, 127). TNF-α imaging was compared to ^18^F-FDG PET and standard laboratory and clinical parameters. Both studies highlighted the variability in TNF-α detection in sarcoidosis patients, as well as the diffuse lung detection compared to higher resolution ^18^F-FDG. This may be due to limitations of scintigraphy vs. PET or represent a complication of imaging soluble TNF-α compared to localized cellular uptake of ^18^F-FDG. A potential alternative could be higher-resolution PET imaging with ^89^Zr or ^111^In labeling to better match the tissue uptake to clearance ratio and half-life of a full-length antibody. Aside from sarcoidosis, TNF-α imaging may also prove useful for diagnostic and monitoring purposes in other TNF-mediated diseases.

Transforming Growth Factor- β (TGF-β) is a cytokine that controls cell proliferation and growth, is often associated with metastatic tumor phenotypes, and is upregulated in glioblastoma, making it a potential treatment target (112, 145). Preclinically, the mAb fresolimumab was labeled with ^89^Zr and imaged by PET in mouse ovarian tumors and human triple negative breast cancer (112). The tracer demonstrated similar distribution to IgG control and lacked specificity to TGF-β-driven tumors, potentially due to the caveat that the mAb only binds the active form of TGF-β. In a clinical study, NCT01472731, ^89^Zr- labeled fresolimumab was used to image patients with recurrent, high-grade gliomas (145). Specificity of the tracer was observed in the brain of all 12 patients, however three lesions larger than 10 mm did not take up the tracer, potentially due to radionecrosis and a lack of viable tissue. Monotherapy with the antibody did not result in an antitumor effect.

In addition to the CD11b tracer discussed above, an interleukin-1 beta (IL-1β) tracer was also tested in the preclinical induced colitis model to detect chronic inflammation (98). Similar to the CD11b tracer, an antibody to IL-1β was ^89^Zr-labeled for PET imaging. Sensitivity of ^89^Zr-anti-IL-1β was similar to the ^89^Zr-anti-CD11b tracer, however biodistribution showed higher specificity to the gastrointestinal tract, with a moderate increase in spleen uptake in colitic mice compared to naïve controls. ^89^Zr-anti-IL-1β exhibited reduced liver and bone marrow uptake compared to ^89^Zr-anti-CD11b. The authors hypothesized ^89^Zr-anti-IL-1β correlated better with colitic severity compared to ^89^Zr-anti-CD11b due to its specificity for activated innate immune cells.

## Directly Labeled Cytokines, Ligands, and Peptides

Interleukin-2 (IL-2) is a cytokine critical for T cell survival and function, and its receptor is highly expressed in activated T cells and Tregs. In a study by Hartimath et al. a radioactively tagged IL-2 tracer, ^18^F-IL-2, was used to target the IL-2 receptor on activated T lymphocytes to monitor response to treatment (113). The authors tested the tracer after treatment with radiation therapy either alone or in combination with a therapeutic human papilloma virus (HPV) vaccine in TC-1 mouse lung tumors, which express the HPV E6 and E7 oncogenes. Tumor uptake of ^18^F-IL-2 was detected after radiation therapy, which was further increased with vaccination. Uptake was reduced after CXCR4 inhibition, which impedes T cell trafficking. Of note, the biodistribution profile of mice treated with radiation plus vaccination showed increased tracer uptake in immune-rich organs such as the spleen, salivary gland, lymph nodes and bone marrow, which is likely due to systemic immune activation. Scintigraphy imaging using ^123^I or ^99m^Tc labeled IL-2 has also been extensively tested in patients to evaluate T cell activity in a variety of immune-related conditions including cancer, Crohn's, atherosclerosis, and type 1 diabetes (21–24, 128). More recently, SPECT was performed with ^99m^Tc-IL-2 in conjunction with MRI to compare uptake in patients with different forms of diabetes. MRI was able to more accurately locate the pancreas for SPECT imaging overlays. This approach identified insulitis and distinguished low-T cell-involvement type 2 diabetes from autoimmune type 1 subsets (129).

Interleukin-12 (IL-12) has a role in various T and NK cell functions, including the induction of IFN-γ, and it is also currently a therapeutic prospect in both cancer for activation of cytolytic cells, as well as chronic hepatitis and colitis, where IL-12 blockade may reduce pathology (146, 147). The IL-12 receptor has been targeted for imaging in a colitis mouse model using ^99m^Tc labeled IL-12 with scintigraphy (114). Tracer accumulation was observed in both normal and diseased tissue with high levels of lymphocytes within 3 h post-injection. There was a detectable level of tracer in the kidney when compared to other radiolabeled cytokines, which may be due to the higher molecular weight of IL-12 (~70 kDa). A caveat to using IL-12 as a tracer is that it is biologically active at doses as low as 300 ng/kg, and thus optimal imaging doses may be toxic or have physiological impact in humans.

**Figure 6:**
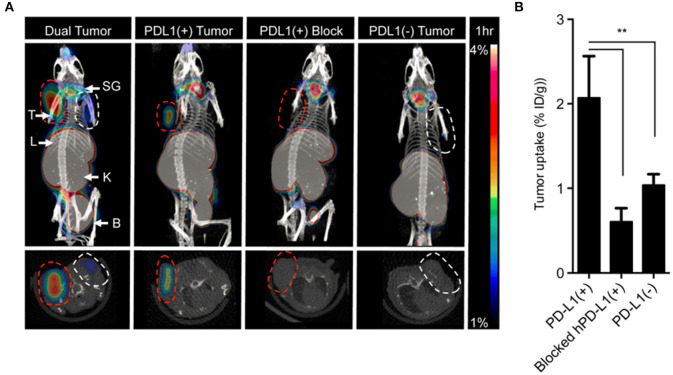
Directly labeled cytokines, ligands and peptides. A high affinity consensus (HAC) of PD-1, ^64^Cu–DOTA–HAC, is an example of a directly labeled ligand that was used to target PD-L1. **(A)** The PET-CT images were acquired 1 h post injection in NSG bearing mice. The specificity of the tracer was evaluated by imaging CT26 tumors that were PD-L1^+^ (red dashed line), PD-L1^−^ (white dashed line), PD-L1^+^ blocked or dual tumors (PD-L1^+^ left, PD-L1^−^ right). **(B)** The uptake was quantified in %ID/g. Error bars represent SD. The decreased uptake in the blocked hPD-L1(+) tumors and the hPD-L1- tumors indicated specificity of the tracer. **P < 0.01. This research was originally published in Maute et al. (115). Permission to reproduce this image has been obtained from the Proceedings of the National Academy of Sciences of the United States of America.

A small molecule tracer was developed using a 14 kDa ectodomain fragment of PD-1 to detect PD-L1 expression. This was tested for PET detection of PD-L1 by radiolabeling with ^64^Cu through a DOTA-maleimide, yielding rapid and specific uptake at 1 h post-injection (Figure 6) (115). An alternative approach is the use of a PD-L1-specific peptide, WL12, selected from a reported library due to its single primary amine for conjugation (118). This tracer has been radiolabeled with ^64^Cu, ^68^Ga, and ^18^F, where it demonstrated specific tumor uptake within 1 h in a variety of immunocompetent and human xenograft mouse models (116–118). Tracer clearance was observed in the liver and kidneys.

A granzyme B-specific PET tracer has also been developed preclinically as a predictive biomarker of response to cancer immunotherapy (119). Because granzyme B is an effector molecule of cytotoxic T and NK cells, it offers a specific imaging option for patients who receive lymphocyte-activating therapies. The tracer is an irreversibly-binding peptide to granzyme B (GZP) containing a flexible glycine-rich linker to permit NOTA conjugation, labeled with short-lived ^68^Ga. Larimer et al. demonstrated the utility of GZP imaging to identify responsive tumors after combination checkpoint blockade therapy in BALB/c mice implanted with syngeneic CT26 colon cancer. Imaging was conducted 1 h post-injection and uptake was observed in all treatment groups in the tumor, kidneys, and bladder, with low uptake levels observed in the treated non-responders and control cohorts. The uptake in the kidneys and bladder was suggested to be consistent with the clearance patterns of small peptide tracers.

## Clinical Perspective

In conventional chemotherapies, radiologic response dictates treatment outcomes, with tumor shrinkage associated with a positive response to treatment. A set criterion was established by the World Health Organization in 1979, which was later replaced with Response Evaluation Criteria in Solid tumors (RECIST) in 2000 (148). A modified version, RECIST 1.1, was adapted in 2009 (149). However, immunotherapy has altered this paradigm with pseudoprogression. In some patients, pseudoprogression occurs when treated tumors appear to progress with an increase in size before subsequently regressing over time. The observed tumor expansion is attributed to a consequence of immune cells infiltrating the lesion as elicited by the treatment. While these morphological changes can be measured through radiological means, it is clinically vital to discriminate pseudoprogression from true progressive disease in order to direct treatment decisions.

To resolve this gap, several guidelines for evaluating radiographic response to immunotherapy were established. Wolchok et al. first reported a set of immune-related response criteria (irRC) adapted from WHO and RECIST guidelines (150). These criteria were borne from observations from ipilimumab-treated patients who experienced tumor progression, particularly in one case where pseudoprogression was displayed as far as at week 12 of tumor assessment. IHC validated the presence of tumor infiltration rather than proliferation. Other immune-related response evaluation criteria have been reported since then with different combinations and variations to irRC and RECIST or RECIST 1.1 (151–153). For a succinct discussion on these guidelines, we refer the readers to the work published by Kataoka et al. (154).

Perhaps of more critical clinical importance is the distinction of whether apparent tumor progression is a consequence of immune infiltration or true progressive disease. Current radiological approaches cannot make this distinction *in situ* and in real time without accompanied histopathology. This validates the argument for developing molecular-based imaging tools to interrogate T cell tumor infiltration and functional status. However, the imaging field has yet to catch up to the exponential rise of immune oncology drugs to monitor response. To the best of our knowledge, there is a dearth of imaging probes that are currently in clinical trials, with no lead agent near FDA approval. This presents a clear and immediate need that should be bridged through concerted efforts among imaging scientists, immunologists, and clinicians to achieve the common goal of successfully eradicating cancer through modulation of immune response.

## Final Conclusions

Reliable imaging technologies for immune monitoring have transformative potential for the management of immune-mediated conditions, particularly with the recent advancement of tumor immunotherapy. Each of the discussed imaging approaches bears a distinct mix of advantages and complications, necessitating careful selection of both target and modality. The goal of this review is to present the current status of this growing field to allow future development of a toolbox of broadly applicable tracers for a variety of conditions. One important consideration to improve the efficacy of immune imaging is validation during development. Although many of the presented tracers are designed for specificity, there remains the potential for non-specific tissue accumulation. Parallel testing with control tracers (e.g., an isotype control for antibody-based tracers) can help indicate the extent of these off-target uptake phenomena, such as EPR effect commonly detected in tumors. Additionally, disease conditions or therapeutics that disrupt vascular homeostasis or integrity can affect tracer accumulation and thus it is important to establish a baseline for these effects. The development of imaging modalities in different disease contexts also warrants additional validation by tissue collection and *ex vivo* analyses to confirm the presence of tracer target.

Due to the dynamic nature of the immune response, careful consideration of the expression kinetics of the target molecule will also be necessary to determine appropriate imaging timing. Following treatment or inflammatory onset, the optimal time point to interrogate tracer uptake compared to controls will need to be established. A final consideration is the potential impact of the tracer on the antigen of interest and immune function through target depletion, neutralization, or modulation of receptor function. It is for this reason that full-length antibody tracers are often fragmented or engineered to eliminate Fc-mediated depletion. Despite these challenges, *in vivo* immune imaging is a promising new frontier with great potential to provide real-time, non-invasive insight into the complex functions of the immune system.

## Author Contributions

CM and JW wrote the manuscript. NV and HG wrote and edited the manuscript.

## Conflict of Interest

The authors declare that the research was conducted in the absence of any commercial or financial relationships that could be construed as a potential conflict of interest.

## References

[1] Chiou VL Burotto M. Pseudoprogression and immune-related response in solid tumors. J Clin Oncol. (2015) 33:3541–3. 10.1200/JCO.2015.61.6870 26261262 PMC4622096

[2] Juergens RA Zukotynski KA Singnurkar A Snider DP Valliant JF Gulenchyn KY. Imaging biomarkers in immunotherapy. Biomark Cancer. (2016) 8:1–13. 10.4137/BIC.S31805 26949344 PMC4768940

[3] Pittet MJ Grimm J Berger CR Tamura T Wojtkiewicz G Nahrendorf M. *In vivo* imaging of T cell delivery to tumors after adoptive transfer therapy. Proc Natl Acad Sci USA. (2007) 104:12457–61. 10.1073/pnas.0704460104 17640914 PMC1941490

[4] McCracken MN Tavaré R Witte ON Wu AM. Advances in PET detection of the antitumor T cell response. Adv Immunol. (2016) 131:187–231. 10.1016/bs.ai.2016.02.004 27235684 PMC5880626

[5] Rahmim A Zaidi H. PET versus SPECT: strengths, limitations and challenges. Nucl Med Commun. (2008) 29:193–207. 10.1097/MNM.0b013e3282f3a515 18349789

[6] Vaquero JJ Kinahan P. Positron emission tomography: current challenges and opportunities for technological advances in clinical and preclinical imaging systems. Annu Rev Biomed Eng. (2015) 17:385–414. 10.1146/annurev-bioeng-071114-040723 26643024 PMC5299095

[7] Reddy S Robinson MK. Immuno-positron emission tomography in cancer models. Semin Nucl Med. (2010) 40:182–9. 10.1053/j.semnuclmed.2009.12.004 20350627 PMC2848398

[8] Kraeber-Bodéré F Rousseau C Bodet-Milin C Mathieu C Guérard F Frampas E. Tumor immunotargeting using innovative radionuclides. Int J Mol Sci. (2015) 16:3932–54. 10.3390/ijms16023932 25679452 PMC4346935

[9] Zhou Y Baidoo KE Brechbiel MW. Mapping biological behaviors by application of longer-lived positron emitting radionuclides. Adv Drug Deliv Rev. (2013) 65:1098–111. 10.1016/j.addr.2012.10.012 23123291 PMC3593806

[10] Stein R Govindan S Hayes M Griffiths GL Hansen HJ Horak ID. Advantage of a residualizing iodine radiolabel in thetherapy of a colon cancer xenograft targeted with an anticarcinoembryonic antigen monoclonal antibody. (2005) 11:2727–34. 10.1158/1078-0432.CCR-04-2100 15814655

[11] Chakraborty S Liu S. 99mTc and 111In-labeling of small biomolecules: bifunctional chelators and related coordination chemistry. Curr Top Med Chem. (2010) 10:1113–34. 10.2174/156802610791384243 20388113

[12] Liu S. Bifunctional coupling agents for radiolabeling of biomolecules and target-specific delivery of metallic radionuclides. Adv Drug Deliv Rev. (2008) 60:1347–70. 10.1016/j.addr.2008.04.006 18538888 PMC2539110

[13] Boschi A Uccelli L Martini P A picture of modern tc-99m radiopharmaceuticals: production, chemistry, and applications in molecular imaging. Appl Sci. (2019) 9:2526 10.3390/app9122526

[14] Liu Y Liu G Hnatowich D A brief review of chelators for radiolabeling oligomers. Materials. (2010) 3:3204–17. 10.3390/ma3053204

[15] Price EW Cawthray JF Bailey GA Ferreira CL Boros E Adam MJ. H4octapa: an acyclic chelator for 111 in radiopharmaceuticals. J Am Chem Soc. (2012). 134:8670–83. 10.1021/ja3024725 22540281

[16] Alirezapour B Jalilian RA Bolourinovin F Moradkhani S. Production and quality control of [(67)ga]-dota-trastuzumab for radioimmunoscintigraphy. Iran J Pharm Res. (2013) 12:355–66. 10.22037/IJPR.2013.1314 24250610 PMC3813249

[17] Brandt M Cardinale J Aulsebrook ML Gasser G Mindt TL. An overview on pet radiochemistry: part 2 - radiometals. J Nucl Med. (2018) 59:1500–6. 10.2967/jnumed.117.190801 29748237

[18] Aluicio-Sarduy E Ellison PA Barnhart TE Cai W Nickles RJ Engle JW. PET radiometals for antibody labeling. J Labelled Comp Radiopharm. (2018). 61:636–51. 10.1002/jlcr.3607 29341227 PMC6050152

[19] Tu Z Mach RH. C-11 Radiochemistry in cancer imaging applications. Curr Top Med Chem. (2010) 10:1060–95. 10.2174/156802610791384261 20388115

[20] Hernandez R Valdovinos HF Yang Y Chakravarty R Hong H Barnhart TE. Sc: an attractive isotope for peptide-based PET imaging. Mol Pharm. (2014) 11:2954–61. 10.1021/mp500343j 25054618 PMC4128785

[21] Annovazzi A Bonanno E Arca M D'Alessandria C Marcoccia A Spagnoli LG. 99mTc-interleukin-2 scintigraphy for the *in vivo* imaging of vulnerable atherosclerotic plaques. Eur J Nucl Med Mol Imaging. (2006) 33:117–26. 10.1007/s00259-005-1899-4 16220305

[22] Chianelli M Parisella MG Visalli N Mather SJ D'Alessandria C Pozzilli P. Pancreatic scintigraphy with 99mTc-interleukin-2 at diagnosis of type 1 diabetes and after 1 year of nicotinamide therapy. Diabetes Metab Res Rev. (2008) 24:115–22. 10.1002/dmrr.767 17918277

[23] Signore A Annovazzi A Barone R Bonanno E D'Alessandria C Chianelli M. 99mTc-interleukin-2 scintigraphy as a potential tool for evaluating tumor-infiltrating lymphocytes in melanoma lesions: a validation study. J Nucl Med. (2004) 45:1647–52. 15471828

[24] Annovazzi A Biancone L Caviglia R Chianelli M Capriotti G Mather SJ. 99mTc-interleukin-2 and 99mTc-HMPAO granulocyte scintigraphy in patients with inactive Crohn's disease. Eur J Nucl Med Mol Imaging. (2003) 30:374–82. 10.1007/s00259-002-1069-x 12634965

[25] Pichler V Berroterán-Infante N Philippe C Vraka C Klebermass E-M Balber T. An overview of PET radiochemistry, part 1: the covalent labels 18F, 11C, and 13N. J Nucl Med. (2018) 59:1350–4. 10.2967/jnumed.117.190793 30042159

[26] Morris O Fairclough M Grigg J Prenant C McMahon A. A review of approaches to 18 F radiolabelling affinity peptides and proteins. J Label Compd Radiopharm. (2019) 62:4–23. 10.1002/jlcr.3634 29740878

[27] Deri MA Zeglis BM Francesconi LC Lewis JS. PET imaging with 89Zr: from radiochemistry to the clinic. Nucl Med Biol. (2013) 40:3–14. 10.1016/j.nucmedbio.2012.08.004 22998840 PMC3517725

[28] Liu J Chen Y Wang G Lv Q Yang Y Wang J Ultrasound molecular imaging of acute cardiac transplantation rejection using nanobubbles targeted to T lymphocytes. Biomaterials. (2018) 162:200–7. 10.1016/j.biomaterials.2018.02.017 29453053

[29] Chen C-L Siow TY Chou C-H Lin C-H Lin M-H Chen Y-C. Targeted superparamagnetic iron oxide nanoparticles for *in vivo* magnetic resonance imaging of T-cells in rheumatoid arthritis. Mol Imaging Biol. (2017) 19:233–44. 10.1007/s11307-016-1001-6 27572293

[30] Jeong Y Hwang HS Na K. Theranostics and contrast agents for magnetic resonance imaging. Biomater Res. (2018) 22:20. 10.1186/s40824-018-0130-1 30065849 PMC6062937

[31] Neuwelt A Sidhu N Hu CAA Mlady G Eberhardt SC Sillerud LO. Iron-based superparamagnetic nanoparticle contrast agents for MRI of infection and inflammation. Am J Roentgenol. (2015) 204:W302–13. 10.2214/AJR.14.12733 25714316 PMC4395032

[32] Sudoł-Szopinska I Cwikła JB. Current imaging techniques in rheumatology: MRI, scintigraphy and PET. Polish J Radiol. (2013) 78:48–56. 10.12659/PJR.889138 24115960 PMC3789933

[33] Ashton JR West JL Badea CT. In vivo small animal micro-CT using nanoparticle contrast agents. Front Pharmacol. (2015) 6:256. 10.3389/fphar.2015.00256 26581654 PMC4631946

[34] Kanwar B Gao DW Hwang AB Grenert JP Williams SP Franc B. *In vivo* imaging of mucosal CD4^+^ T cells using single photon emission computed tomography in a murine model of colitis. J Immunol Methods. (2008) 329:21–30. 10.1016/j.jim.2007.09.008 17964595 PMC2683264

[35] Piccinelli M Garcia EV. Advances in single-photon emission computed tomography hardware and software. Cardiol Clin. (2016) 34:1–11. 10.1016/j.ccl.2015.06.001 26590775

[36] Dimitrakopoulou-Strauss A. Monitoring of patients with metastatic melanoma treated with immune checkpoint inhibitors using PET-CT. Cancer Immunol Immunother. (2019) 68:813–22. 10.1007/s00262-018-2229-6 30123922 PMC11028039

[37] Glaudemans AWJM de Vries EFJ Galli F Dierckx RAJO Slart RHJA Signore A. The use of (18)F-FDG-PET/CT for diagnosis and treatment monitoring of inflammatory and infectious diseases. Clin Dev Immunol. (2013) 2013:623036. 10.1155/2013/623036 24027590 PMC3763592

[38] Shah NN Nagle SJ Torigian DA Farwell MD Hwang W-T Frey N. Early positron emission tomography/computed tomography as a predictor of response after CTL019 chimeric antigen receptor -T-cell therapy in B-cell non-Hodgkin lymphomas. Cytotherapy. (2018) 20:1415–8. 10.1016/j.jcyt.2018.10.003 30385043

[39] Chargari C Le Moulec S Bonardel G Foehrenbach H Védrine L. Ipilimumab in cancer patients. Anticancer Drugs. (2013) 24:324–6. 10.1097/CAD.0b013e32835dbaaf 23348246

[40] Zaucha JM Chauvie S Zaucha R Biggii A Gallamini A. The role of PET/CT in the modern treatment of hodgkin lymphoma. Cancer Treat Rev. (2019) 77:44–56. 10.1016/j.ctrv.2019.06.002 31260900

[41] Chrapko BE Chrapko M Nocun A Stefaniak B Zubilewicz T Drop A. Role of 18F-FDG PET/CT in the diagnosis of inflammatory and infectious vascular disease. Nucl Med Rev Cent East Eur. (2016) 19:28–36. 10.5603/NMR.2016.0006 26841377

[42] Shrot S Schachter J Shapira-Frommer R Besser MJ Apter S. CT halo sign as an imaging marker for response to adoptive cell therapy in metastatic melanoma with pulmonary metastases. Eur Radiol. (2014) 24:1251–6. 10.1007/s00330-014-3129-6 24663820

[43] Li D Li X Zhou W-L Huang Y Liang X Jiang L Genetically engineered T cells for cancer immunotherapy. Signal Transduct Target Ther. (2019) 4:35 10.1038/s41392-019-0070-9 31637014 PMC6799837

[44] Meidenbauer N Marienhagen J Laumer M Vogl S Heymann J Andreesen R. Survival and tumor localization of adoptively transferred melan-A-specific T cells in melanoma patients. J Immunol. (2003) 170:2161–9. 10.4049/jimmunol.170.4.2161 12574389

[45] Parente-Pereira AC Burnet J Ellison D Foster J Davies DM van der Stegen S Trafficking of CAR-engineered human T cells following regional or systemic adoptive transfer in SCID beige mice. J Clin Immunol. (2011) 31:710–18. 10.1007/s10875-011-9532-8 21505816

[46] Bernhard H Neudorfer J Gebhard K Conrad H Hermann C Nährig J. Adoptive transfer of autologous, HER2-specific, cytotoxic T lymphocytes for the treatment of HER2-overexpressing breast cancer. Cancer Immunol Immunother. (2007) 57:271–80. 10.1007/s00262-007-0355-7 17646988 PMC11030865

[47] Stanton SE Eary JF Marzbani EA Mankoff D Salazar LG Higgins D. Concurrent SPECT/PET-CT imaging as a method for tracking adoptively transferred T-cells *in vivo*. J Immunother Cancer. (2016) 4:27. 10.1186/s40425-016-0131-3 27190628 PMC4869363

[48] Grabner A Kentrup D Edemir B Sirin Y Pavenstädt H Schlatter E. PET with 18F-FDG-labeled T lymphocytes for diagnosis of acute rat renal allograft rejection. J Nucl Med. (2013) 54:1147–53. 10.2967/jnumed.112.109231 23670903

[49] Bansal A Pandey MK Demirhan YE Nesbitt JJ Crespo-Diaz RJ Terzic A. Novel (89)Zr cell labeling approach for PET-based cell trafficking studies. EJNMMI Res. (2015) 5:19. 10.1186/s13550-015-0098-y 25918673 PMC4401478

[50] Weist MR Starr R Aguilar B Chea J Miles JK Poku E. PET of adoptively transferred chimeric antigen receptor T Cells with 89Zr-Oxine. J Nucl Med. (2018) 59:1531–7. 10.2967/jnumed.117.206714 29728514 PMC6167529

[51] Charoenphun P Meszaros LK Chuamsaamarkkee K Sharif-Paghaleh E Ballinger JR Ferris TJ. [89Zr]Oxinate4 for long-term *in vivo* cell tracking by positron emission tomography. Eur J Nucl Med Mol Imaging. (2015) 42:278–87. 10.1007/s00259-014-2945-x 25359636 PMC4315484

[52] Ferris TJ Charoenphun P Meszaros LK Mullen GED Blower PJ Went MJ. Synthesis and characterisation of zirconium complexes for cell tracking with Zr-89 by positron emission tomography. Dalton Trans. (2014) 43:14851–7. 10.1039/C4DT01928H 25164373 PMC6205629

[53] Sato N Wu H Asiedu KO Szajek LP Griffiths GL Choyke PL. (89)Zr-oxine complex PET cell imaging in monitoring cell-based therapies. Radiology. (2015) 275:490–500. 10.1148/radiol.15142849 25706654 PMC4456181

[54] Asiedu KO Koyasu S Szajek LP Choyke PL Sato N. Bone marrow cell trafficking analyzed by 89zr-oxine positron emission tomography in a murine transplantation model. Clin Cancer Res. (2017) 23:2759–68. 10.1158/1078-0432.CCR-16-1561 27965305 PMC5457332

[55] Berg E Gill H Marik J Ogasawara A Williams SP van Dongen GAMS. Total-body PET and highly stable chelators together enable meaningful 89 Zr-antibody-PET studies up to 30 days post-injection . J Nucl Med. (2019) 61:453–60. 10.2967/jnumed.119.230961 31562219 PMC7067524

[56] El Ansary M Mogawer S Elhamid SA Alwakil S Aboelkasem F El Sabaawy H. Immunotherapy by autologous dendritic cell vaccine in patients with advanced HCC. J Cancer Res Clin Oncol. (2013) 139:39–48. 10.1007/s00432-012-1298-8 22886490 PMC5223882

[57] Andrés C Plana M Guardo AC Alvarez-Fernández C Climent N Gallart T. HIV-1 reservoir dynamics after vaccination and antiretroviral therapy interruption are associated with dendritic cell vaccine-induced t cell responses. J Virol. (2015) 89:9189–99. 10.1128/JVI.01062-15 26109727 PMC4542373

[58] Li C Liang S Zhang C Liu Y Yang M Zhang J. Allogenic dendritic cell and tumor cell fused vaccine for targeted imaging and enhanced immunotherapeutic efficacy of gastric cancer. Biomaterials. (2015) 54:177–87. 10.1016/j.biomaterials.2015.03.024 25907051

[59] Rosenblatt J Glotzbecker B Mills H Vasir B Tzachanis D Levine JD. PD-1 blockade by CT-011, anti-PD-1 antibody, enhances *ex vivo* T-cell responses to autologous dendritic cell/myeloma fusion vaccine. J Immunother. (2011) 34:409–18. 10.1097/CJI.0b013e31821ca6ce 21577144 PMC3142955

[60] Zhang Z Li W Procissi D Li K Sheu AY Gordon AC. Antigen-loaded dendritic cell migration: mr imaging in a pancreatic carcinoma model. Radiology. (2015) 274:192–200. 10.1148/radiol.14132172 25222066 PMC4314117

[61] Xu Y Wu C Zhu W Xia C Wang D Zhang H. Superparamagnetic MRI probes for *in vivo* tracking of dendritic cell migration with a clinical 3 T scanner. Biomaterials. (2015) 58:63–71. 10.1016/j.biomaterials.2015.04.016 25941783

[62] Ferguson PM Slocombe A Tilley RD Hermans IF. Using magnetic resonance imaging to evaluate dendritic cell-based vaccination. PLoS ONE. (2013) 8:e65318. 10.1371/journal.pone.0065318 23734246 PMC3667033

[63] Serkova NJ. Nanoparticle-based magnetic resonance imaging on tumor-associated macrophages and inflammation. Front Immunol. (2017) 8:590. 10.3389/fimmu.2017.00590 28588582 PMC5439008

[64] Kanno S Lee PC Dodd SJ Williams M Griffith BP Ho C. A novel approach with magnetic resonance imaging used for the detection of lung allograft rejection. J Thorac Cardiovasc Surg. (2000) 120:923–34. 10.1067/mtc.2000.110184 11044319

[65] Weissleder R Nahrendorf M Pittet MJ Imaging macrophages with nanoparticles. Nat Mater. (2014) 13:125–38. 10.1038/nmat3780 24452356

[66] Yeh TC Zhang W Ildstad ST Ho C. *In vivo* dynamic MRI tracking of rat T-cells labeled with superparamagnetic iron-oxide particles. Magn Reson Med. (1995) 33:200–8. 10.1002/mrm.1910330209 7707910

[67] Yeh TC Zhang W Ildstad ST Ho C. Intracellular labeling of T-cells with superparamagnetic contrast agents. Magn Reson Med. (1993) 30:617–25. 10.1002/mrm.1910300513 8259062

[68] Eisenstein S Coakley BA Briley-Saebo K Ma G Chen H-M Meseck M. Myeloid-derived suppressor cells as a vehicle for tumor-specific oncolytic viral therapy. Cancer Res. (2013) 73:5003–15. 10.1158/0008-5472.CAN-12-1597 23536556 PMC3745543

[69] Yaghoubi SS Gambhir SS PET imaging of herpes simplex virus type 1 thymidine kinase (HSV1-tk) or mutant HSV1-sr39tk reporter gene expression in mice and humans using [18F]FHBG. Nat Protoc. (2006) 1:3069–75. 10.1038/nprot.2006.459 17406570

[70] Likar Y Zurita J Dobrenkov K Shenker L Cai S Neschadim A. A new pyrimidine-specific reporter gene: a mutated human deoxycytidine kinase suitable for PET during treatment with acycloguanosine-based cytotoxic drugs. J Nucl Med. (2010) 51:1395–403. 10.2967/jnumed.109.074344 20810757 PMC4405132

[71] McCracken MN Vatakis DN Dixit D McLaughlin J Zack JA Witte ON. Noninvasive detection of tumor-infiltrating T cells by PET reporter imaging. J Clin Invest. (2015) 125:1815–26. 10.1172/JCI77326 25822024 PMC4463193

[72] Sellmyer MA Richman SA Lohith K Hou C Weng C-C Mach RH. Imaging CAR T cell trafficking with eDHFR as a PET reporter gene. Mol Ther. (2020) 28:42–51. 10.1016/j.ymthe.2019.10.007 31668558 PMC6953896

[73] Emami-Shahri N Foster J Kashani R Gazinska P Cook C Sosabowski J. Clinically compliant spatial and temporal imaging of chimeric antigen receptor T-cells. Nat Commun. (2018) 9:1081. 10.1038/s41467-018-03524-1 29540684 PMC5852048

[74] Sharif-Paghaleh E Sunassee K Tavaré R Ratnasothy K Koers A Ali N. *In vivo* SPECT reporter gene imaging of regulatory T cells. PLoS ONE. (2011) 6:e25857. 10.1371/journal.pone.0025857 22043296 PMC3197183

[75] Ahn S-B Lee SB Singh TD Cho SJ Kim SK Lee I-K. Multimodality imaging of bone marrow-derived dendritic cell migration and antitumor immunity. Transl Oncol. (2017) 10:262–70. 10.1016/j.tranon.2017.01.003 28214774 PMC5314440

[76] Lee SB Lee HW Lee H Jeon YH Lee S-W Ahn B-C. Tracking dendritic cell migration into lymph nodes by using a novel PET probe 18F-tetrafluoroborate for sodium/iodide symporter. EJNMMI Res. (2017) 7:32. 10.1186/s13550-017-0280-5 28378292 PMC5380646

[77] Lee H Lee HW La Lee Y Jeon YH Jeong SY Lee S-W. Optimization of dendritic cell-mediated cytotoxic T-cell activation by tracking of dendritic cell migration using reporter gene imaging. Mol imaging Biol. (2018) 20:398–406. 10.1007/s11307-017-1127-1 29027077

[78] Doubrovin MM Doubrovina ES Zanzonico P Sadelain M Larson SM O'Reilly RJ. *In vivo* imaging and quantitation of adoptively transferred human antigen-specific T cells transduced to express a human norepinephrine transporter gene. Cancer Res. (2007) 67:11959–69. 10.1158/0008-5472.CAN-07-1250 18089827

[79] Moroz MA Zhang H Lee J Moroz E Zurita J Shenker L. Comparative analysis of T cell imaging with human nuclear reporter genes. J Nucl Med. (2015) 56:1055–60. 10.2967/jnumed.115.159855 26025962 PMC4511596

[80] Krebs S Ahad A Carter LM Eyquem J Brand C Bell M. Antibody with infinite affinity for *in vivo* tracking of genetically engineered lymphocytes. J Nucl Med. (2018) 59:1894–900. 10.2967/jnumed.118.208041 29903928 PMC6278895

[81] Minn I Huss DJ Ahn H-H Chinn TM Park A Jones J. Imaging CAR T cell therapy with PSMA-targeted positron emission tomography. Sci Adv. (2019) 5:eaaw5096. 10.1126/sciadv.aaw5096 31281894 PMC6609218

[82] Beckford Vera DR Smith CC Bixby LM Glatt DM Dunn SS Saito R. Immuno-PET imaging of tumor-infiltrating lymphocytes using zirconium-89 radiolabeled anti-CD3 antibody in immune-competent mice bearing syngeneic tumors. PLoS ONE. (2018) 13:e0193832. 10.1371/journal.pone.0193832 29513764 PMC5841805

[83] Larimer BM Wehrenberg-Klee E Caraballo A Mahmood U. Quantitative CD3 PET imaging predicts tumor growth response to anti-CTLA-4 therapy. J Nucl Med. (2016) 57:1607–11. 10.2967/jnumed.116.173930 27230929 PMC5367446

[84] Gibson HM McKnight BN Malysa A Dyson G Wiesend WN McCarthy CE. IFNγ PET imaging as a predictive tool for monitoring response to tumor immunotherapy. Cancer Res. (2018) 78:5706–17. 10.1158/0008-5472.CAN-18-0253 30115693 PMC6443251

[85] Malviya G D'Alessandria C Bonanno E Vexler V Massari R Trotta C. Radiolabeled humanized anti-CD3 monoclonal antibody visilizumab for imaging human T-lymphocytes. J Nucl Med. (2009) 50:1683–91. 10.2967/jnumed.108.059485 19759100

[86] Martins FPP Souza SAL Gonçalves RT Fonseca LMB Gutfilen B. Preliminary results of [99mTc]OKT3 scintigraphy to evaluate acute rejection in renal transplants. Transplant Proc. (2004) 36:2664–7. 10.1016/j.transproceed.2004.09.085 15621118

[87] Grabner A Kentrup D Mühlmeister M Pawelski H Biermann C Bettinger T. Noninvasive imaging of acute renal allograft rejection by ultrasound detection of microbubbles targeted to T-lymphocytes in rats. Ultraschall Medizin Eur J Ultrasound. (2015) 37:82–91. 10.1055/s-0034-1385796 25919412

[88] Mayer KE Mall S Yusufi N Gosmann D Steiger K Russelli L. T-cell functionality testing is highly relevant to developing novel immuno-tracers monitoring T cells in the context of immunotherapies and revealed CD7 as an attractive target. Theranostics. (2018) 8:6070–87. 10.7150/thno.27275 30613283 PMC6299443

[89] Tavaré R McCracken MN Zettlitz KA Knowles SM Salazar FB Olafsen T. Engineered antibody fragments for immuno-PET imaging of endogenous CD8^+^ T cells *in vivo*. Proc Natl Acad Sci USA. (2014) 111:1108–13. 10.1073/pnas.1316922111 24390540 PMC3903195

[90] Tavaré R Escuin-Ordinas H Mok S McCracken MN Zettlitz KA Salazar FB. An effective immuno-PET imaging method to monitor CD8-dependent responses to immunotherapy. Cancer Res. (2016) 76:73–82. 10.1158/0008-5472.CAN-15-1707 26573799 PMC4703530

[91] Freise AC Zettlitz KA Salazar FB Tavaré R Tsai W-TK Chatziioannou AF. Immuno-PET in inflammatory bowel disease: imaging CD4-positive T cells in a murine model of colitis. J Nucl Med. (2018) 59:980–5. 10.2967/jnumed.117.199075 29326360 PMC6004558

[92] Freise AC Zettlitz KA Salazar FB Lu X Tavaré R Wu AM. ImmunoPET Imaging of Murine CD4^+^ T cells using anti-CD4 cys-diabody: effects of protein dose on T cell function and imaging. Mol Imaging Biol. (2017) 19:599–609. 10.1007/s11307-016-1032-z 27966069 PMC5524218

[93] Yoon JT Longtine MS Marquez-Nostra BV Wahl RL. Evaluation of next-generation anti-CD20 antibodies labeled with 89 Zr in human lymphoma xenografts. J Nucl Med. (2018) 59:1219–24. 10.2967/jnumed.117.203299 29348316 PMC6071500

[94] Natarajan A Hackel BJ Gambhir SS. A novel engineered anti-CD20 tracer enables early time PET imaging in a humanized transgenic mouse model of B-cell non-hodgkins lymphoma. Clin Cancer Res. (2013) 19:6820–9. 10.1158/1078-0432.CCR-13-0626 24097872

[95] Zettlitz KA Tavaré R Knowles SM Steward KK Timmerman JM Wu AM. ImmunoPET of malignant and normal B Cells with 89Zr- and 124I-Labeled obinutuzumab antibody fragments reveals differential CD20 internalization *in vivo*. Clin Cancer Res. (2017) 23:7242–52. 10.1158/1078-0432.CCR-17-0855 28928164 PMC5880625

[96] Zettlitz KA Tavaré R Tsai W-TK Yamada RE Ha NS Collins J. 18F-labeled anti-human CD20 cys-diabody for same-day immunoPET in a model of aggressive B cell lymphoma in human CD20 transgenic mice. Eur J Nucl Med Mol Imaging. (2019) 46:489–500. 10.1007/s00259-018-4214-x 30456475 PMC6580847

[97] Stevens M Cropper H Jackson I Chaney A Lechtenberg K Buckwalter M Radiolabeling and pre-clinical evaluation of a first-in-class CD19 PET Tracer for imaging B cells in multiple sclerosis. J Nucl Med. (2019) 60:129.30213846

[98] Dmochowska N Tieu W Keller MD Wardill HR Mavrangelos C Campaniello MA. Immuno-PET of innate immune markers CD11b and IL-1β detects inflammation in murine colitis. J Nucl Med. (2019) 60:858–63. 10.2967/jnumed.118.219287 30413657 PMC6581233

[99] Chatterjee S Lesniak WG Gabrielson M Lisok A Wharram B Sysa-Shah P. A humanized antibody for imaging immune checkpoint ligand PD-L1 expression in tumors. Oncotarget. (2016) 7:10215–27. 10.18632/oncotarget.7143 26848870 PMC4891115

[100] Heskamp S Hobo W Molkenboer-Kuenen JDM Olive D Oyen WJG Dolstra H. Noninvasive imaging of tumor PD-L1 expression using radiolabeled anti-PD-L1 antibodies. Cancer Res. (2015) 75:2928–36. 10.1158/0008-5472.CAN-14-3477 25977331

[101] Josefsson A Nedrow JR Park S Banerjee SR Rittenbach A Jammes F. Imaging, biodistribution, and dosimetry of radionuclide-labeled PD-L1 antibody in an immunocompetent mouse model of breast cancer. Cancer Res. (2016) 76:472–9. 10.1158/0008-5472.CAN-15-2141 26554829 PMC4715915

[102] Chatterjee S Lesniak WG Nimmagadda S. Noninvasive imaging of immune checkpoint ligand PD-L1 in tumors and metastases for guiding immunotherapy. Mol Imaging. (2017) 16:153601211771845. 10.1177/1536012117718459 28707500 PMC5676497

[103] Lesniak WG Chatterjee S Gabrielson M Lisok A Wharram B Pomper MG. PD-L1 detection in tumors using [(64)Cu]Atezolizumab with PET. Bioconjug Chem. (2016) 27:2103–10. 10.1021/acs.bioconjchem.6b00348 27458027 PMC5289227

[104] Ingram JR Dougan M Rashidian M Knoll M Keliher EJ Garrett S. PD-L1 is an activation-independent marker of brown adipocytes. Nat Commun. (2017) 8:647. 10.1038/s41467-017-00799-8 28935898 PMC5608754

[105] Li D Cheng S Zou S Zhu D Zhu T Wang P. Immuno-PET imaging of 89Zr labeled anti-PD-L1 domain antibody. Mol Pharm. (2018) 15:1674–81. 10.1021/acs.molpharmaceut.8b00062 29502426

[106] Hettich M Braun F Bartholomä MD Schirmbeck R Niedermann G. High-Resolution PET imaging with therapeutic antibody-based PD-1/PD-L1 checkpoint tracers. Theranostics. (2016) 6:1629–40. 10.7150/thno.15253 27446497 PMC4955062

[107] Natarajan A Mayer AT Xu L Reeves RE Gano J Gambhir SS. Novel radiotracer for immunoPET Imaging of PD-1 Checkpoint expression on tumor infiltrating lymphocytes. Bioconjug Chem. (2015) 26:2062–69. 10.1021/acs.bioconjchem.5b00318 26307602

[108] Natarajan A Patel CB Habte F Gambhir SS. Dosimetry prediction for clinical translation of 64Cu-Pembrolizumab immunoPET targeting human PD-1 expression. Sci Rep. (2018) 8:633. 10.1038/s41598-017-19123-x 29330552 PMC5766550

[109] Higashikawa K Yagi K Watanabe K Kamino S Ueda M Hiromura M. 64Cu-DOTA-anti-CTLA-4 mAb enabled PET visualization of CTLA-4 on the T-cell infiltrating tumor tissues. PLoS ONE. (2014) 9:e109866. 10.1371/journal.pone.0109866 25365349 PMC4217715

[110] Ehlerding EB England CG Majewski RL Valdovinos HF Jiang D Liu G. ImmunoPET imaging of CTLA-4 expression in mouse models of non-small cell lung cancer. Mol Pharm. (2017) 14:1782–9. 10.1021/acs.molpharmaceut.7b00056 28388076 PMC5495656

[111] Alam IS Mayer AT Sagiv-Barfi I Wang K Vermesh O Czerwinski DK. Imaging activated T cells predicts response to cancer vaccines. J Clin Invest. (2018) 128:2569–80. 10.1172/JCI98509 29596062 PMC5983309

[112] Oude Munnink TH Arjaans MEA Timmer-Bosscha H Schröder CP Hesselink JW Vedelaar SR. PET with the 89Zr-labeled transforming growth factor-β antibody fresolimumab in tumor models. J Nucl Med. (2011) 52:2001–8. 10.2967/jnumed.111.092809 22072706

[113] Hartimath SV Draghiciu O van de Wall S Manuelli V Dierckx RAJO Nijman HW. Noninvasive monitoring of cancer therapy induced activated T cells using [18 F]FB-IL-2 PET imaging. Oncoimmunology. (2017) 6:e1248014. 10.1080/2162402X.2016.1248014 28197364 PMC5283633

[114] Annovazzi A D'Alessandria C Bonanno E Mather SJ Cornelissen B van de Wiele C. Synthesis of 99mTc-HYNIC-interleukin-12, a new specific radiopharmaceutical for imaging T lymphocytes. Eur J Nucl Med Mol Imaging. (2006) 33:474–82. 10.1007/s00259-005-0001-6 16619112

[115] Maute RL Gordon SR Mayer AT McCracken MN Natarajan A Ring NG. Engineering high-affinity PD-1 variants for optimized immunotherapy and immuno-PET imaging. Proc Natl Acad Sci USA. (2015) 112:E6506–14. 10.1073/pnas.1519623112 26604307 PMC4664306

[116] De Silva RA Kumar D Lisok A Chatterjee S Wharram B Venkateswara Rao K. Peptide-Based 68 Ga-PET radiotracer for imaging PD-L1 expression in cancer. Mol Pharm. (2018) 15:3946–52. 10.1021/acs.molpharmaceut.8b00399 30037229 PMC6127800

[117] Lesniak WG Mease RC Chatterjee S Kumar D Lisok A Wharram B. Development of [18 F]FPy-WL12 as a PD-L1 specific PET imaging peptide. Mol Imaging. (2019) 18:153601211985218. 10.1177/1536012119852189 31187691 PMC6563393

[118] Chatterjee S Lesniak WG Miller MS Lisok A Sikorska E Wharram B. Rapid PD-L1 detection in tumors with PET using a highly specific peptide. Biochem Biophys Res Commun. (2017) 483:258–63. 10.1016/j.bbrc.2016.12.156 28025143 PMC5253331

[119] Larimer BM Wehrenberg-Klee E Dubois F Mehta A Kalomeris T Flaherty K. Granzyme B PET imaging as a predictive biomarker of immunotherapy response. Cancer Res. (2017) 77:2318–27. 10.1158/0008-5472.CAN-16-3346 28461564 PMC5474226

[120] Yaghoubi SS Jensen MC Satyamurthy N Budhiraja S Paik D Czernin J. Noninvasive detection of therapeutic cytolytic T cells with 18F-FHBG PET in a patient with glioma. Nat Clin Pract Oncol. (2008) 6:53–8. 10.1038/ncponc1278 19015650 PMC3526373

[121] Keu KV Witney TH Yaghoubi S Rosenberg J Kurien A Magnusson R. Reporter gene imaging of targeted T cell immunotherapy in recurrent glioma. Sci Transl Med. (2017) 9:eaag2196. 10.1126/scitranslmed.aag2196 28100832 PMC5260938

[122] Lopes FP de Azevedo MN Marchiori E da Fonseca LM de Souza SA Gutfilen B. Use of 99mTc-anti-CD3 scintigraphy in the differential diagnosis of rheumatic diseases. Rheumatology. (2010) 49:933–9. 10.1093/rheumatology/kep471 20129997

[123] Martins FPP Gutfilen B de Souza SAL de Azevedo MNL Cardoso LR Fraga R. Monitoring rheumatoid arthritis synovitis with 99m Tc-anti-CD3. Br J Radiol. (2008) 81:25–9. 10.1259/bjr/63780400 18039720

[124] Marcus C Thakur ML Huynh TV Louie JS Leibling M Minami C. Imaging rheumatic joint diseases with anti-T lymphocyte antibody OKT-3. Nucl Med Commun. (1994) 15:824–30. 10.1097/00006231-199410000-00008 7838446

[125] Tran L Huitema ADR van Rijswijk MH Dinant HJ Baars JW Beijnen JH CD20 antigen imaging with 124I-rituximab PET/CT in patients with rheumatoid arthritis. Hum Antibodies. (2011) 20:29–35. 10.3233/HAB-2011-0239 21558621

[126] Vis R Malviya G Signore A Grutters JC Meek B van de Garde EMW. 99mTc-anti-TNF-α antibody for the imaging of disease activity in pulmonary sarcoidosis. Eur Respir J. (2016) 47:1198–207. 10.1183/13993003.01352-2015 26797030

[127] Galli F Lanzolla T Pietrangeli V Malviya G Ricci A Bruno P. *In vivo* evaluation of TNF-alpha in the lungs of patients affected by sarcoidosis. Biomed Res Int. (2015) 2015:401341. 10.1155/2015/401341 25866780 PMC4383433

[128] Signore A Chianelli M Annovazzi A Bonanno E Spagnoli LG Pozzilli P. 123I-interleukin-2 scintigraphy for *in vivo* assessment of intestinal mononuclear cell infiltration in Crohn's disease. J Nucl Med. (2000) 41:242–9. 10688106

[129] Signore A Capriotti G Chianelli M Bonanno E Galli F Catalano C. Detection of insulitis by pancreatic scintigraphy with 99mTc-labeled IL-2 and MRI in patients with LADA (Action LADA 10). Diabetes Care. (2015) 38:652–8. 10.2337/dc14-0580 25665813

[130] Vedvyas Y Shevlin E Zaman M Min IM Amor-Coarasa A Park S. Longitudinal PET imaging demonstrates biphasic CAR T cell responses in survivors. JCI insight. (2016) 1:e90064. 10.1172/jci.insight.90064 27882353 PMC5111513

[131] Freise AC Wu AM. *In vivo* imaging with antibodies and engineered fragments. Mol Immunol. (2015) 67:142–52. 10.1016/j.molimm.2015.04.001 25934435 PMC4529772

[132] Wold ED Smider VV Felding BH. Antibody Therapeutics in Oncology. Immunother. (2016) 2:108–117. 10.4172/2471-9552.1000108 27081677 PMC4829403

[133] Wu AM Engineered antibodies for molecular imaging of cancer. Methods. (2014) 65:139–47. 10.1016/j.ymeth.2013.09.015 24091005 PMC3947235

[134] Ryman JT Meibohm B. Pharmacokinetics of Monoclonal Antibodies. CPT Pharmacometrics Syst Pharmacol. (2017) 6:576–88. 10.1002/psp4.12224 28653357 PMC5613179

[135] Severin GW Jørgensen JT Wiehr S Rolle A-M Hansen AE Maurer A. The impact of weakly bound 89Zr on preclinical studies: non-specific accumulation in solid tumors and aspergillus infection. Nucl Med Biol. (2015) 42:360–8. 10.1016/j.nucmedbio.2014.11.005 25583221

[136] Holland JP Sheh Y Lewis JS. Standardized methods for the production of high specific-activity zirconium-89. Nucl Med Biol. (2009) 36:729–39. 10.1016/j.nucmedbio.2009.05.007 19720285 PMC2827875

[137] Chitnis T. The role of CD4 T cells in the pathogenesis of multiple sclerosis. Int Rev Neurobiol. (2007) 79:43–72. 10.1016/S0074-7742(07)79003-7 17531837 PMC7112308

[138] Imam T Park S Kaplan MH Olson MR. Effector T helper cell subsets in inflammatory bowel diseases. Front Immunol. (2018) 9:1212. 10.3389/fimmu.2018.01212 29910812 PMC5992276

[139] Du FH Mills EA Mao-Draayer Y. Next-generation anti-CD20 monoclonal antibodies in autoimmune disease treatment. Auto Immun Highlights. (2017) 8:12. 10.1007/s13317-017-0100-y 29143151 PMC5688039

[140] Maloney DG. Immunotherapy for non-hodgkin's lymphoma: monoclonal antibodies and vaccines. J Clin Oncol. (2005) 23:6421–8. 10.1200/JCO.2005.06.004 16155029

[141] Muylle K Flamen P Vugts DJ Guiot T Ghanem G Meuleman N. Tumour targeting and radiation dose of radioimmunotherapy with 90Y-rituximab in CD20^+^ B-cell lymphoma as predicted by 89Zr-rituximab immuno-PET: impact of preloading with unlabelled rituximab. Eur J Nucl Med Mol Imaging. (2015) 42:1304–14. 10.1007/s00259-015-3025-6 25792453 PMC4480335

[142] Rahmanzadeh R Weber MS Brück W Navardi S Sahraian MA. B cells in multiple sclerosis therapy-A comprehensive review. Acta Neurol Scand. (2018) 137:544–56. 10.1111/ane.12915 29512131

[143] Zhang M Jiang H Zhang R Jiang H Xu H Pan W. Near-infrared fluorescence-labeled anti-PD-L1-mAb for tumor imaging in human colorectal cancer xenografted mice. J Cell Biochem. (2019) 120:10239–47. 10.1002/jcb.28308 30609118 PMC6590288

[144] Atzeni F Sarziputtini P Doria A Iaccarino L Capsoni F. Potential off-label use of infliximab in autoimmune and non-autoimmune diseases: a review. Autoimmun Rev. (2005) 4:144–52. 10.1016/j.autrev.2004.08.004 15823500

[145] den Hollander MW Bensch F Glaudemans AWJM Oude Munnink TH Enting RH den Dunnen WFA. TGF-β antibody uptake in recurrent high-grade glioma imaged with 89Zr-Fresolimumab PET. J Nucl Med. (2015) 56:1310–4. 10.2967/jnumed.115.154401 26135113

[146] Wang P Li X Wang J Gao D Li Y Li H. Re-designing interleukin-12 to enhance its safety and potential as an anti-tumor immunotherapeutic agent. Nat Commun. (2017) 8:1395. 10.1038/s41467-017-01385-8 29123084 PMC5680234

[147] Lee HS Park S-K Park D Il. Novel treatments for inflammatory bowel disease. Korean J Intern Med. (2018) 33:20–7. 10.3904/kjim.2017.393 29223139 PMC5768553

[148] Suzuki C Jacobsson H Hatschek T Torkzad MR Bodén K Eriksson-Aim Y. Radiologic measurements of tumor response to treatment: practical approaches and limitations. Radiographics. (2008) 28:329–44. 10.1148/rg.282075068 18349443

[149] Eisenhauer EA Therasse P Bogaerts J Schwartz LH Sargent D Ford R. New response evaluation criteria in solid tumours: revised RECIST guideline (version 1.1). Eur J Cancer. (2009) 45:228–47. 10.1016/j.ejca.2008.10.026 19097774

[150] Wolchok JD Hoos A O'Day S Weber JS Hamid O Lebbé C. Guidelines for the evaluation of immune therapy activity in solid tumors: immune-related response criteria. Clin Cancer Res. (2009) 15:7412–20. 10.1158/1078-0432.CCR-09-1624 19934295

[151] Nishino M Giobbie-Hurder A Gargano M Suda M Ramaiya NH Hodi FS. Developing a common language for tumor response to immunotherapy: immune-related response criteria using unidimensional measurements. Clin Cancer Res. (2013) 19:3936–43. 10.1158/1078-0432.CCR-13-0895 23743568 PMC3740724

[152] Seymour L Bogaerts J Perrone A Ford R Schwartz LH Mandrekar S. iRECIST: guidelines for response criteria for use in trials testing immunotherapeutics. Lancet Oncol. (2017) 18:e143–52. 10.1016/S1470-2045(17)30074-8 28271869 PMC5648544

[153] Stephen Hodi F Ballinger M Lyons B Soria JC Nishino M Tabernero J. Immune-modified response evaluation criteria in solid tumors (imrecist): refining guidelines to assess the clinical benefit of cancer immunotherapy. J Clin Oncol. (2018) 36:850–8. 10.1200/JCO.2017.75.1644 29341833

[154] Kataoka Y Hirano K. Which criteria should we use to evaluate the efficacy of immune-checkpoint inhibitors? Ann Transl Med. (2018) 6:222. 10.21037/atm.2018.04.17 30023385 PMC6035987

